# FRIENDS – friendship-based regulation of individual emotions in networks in daily situations: An EMA study on partner–motive pairing and social outcomes in adult friendships

**DOI:** 10.1186/s40359-026-05767-w

**Published:** 2026-10-14

**Authors:** Paula Schott, Katharina Enser, Martin Weiß

**Affiliations:** Department of Psychology I: Clinical Psychology and Psychotherapy, Institute of Psychology, University of Würzburg, Würzburg, Germany

**Keywords:** Interpersonal emotion regulation, Friendship network, Ecological momentary assessment, Social loneliness, Psychological well-being

## Abstract

**Background:**

Interpersonal emotion regulation depends not only on which strategy is used but on whom people turn to. Previous work suggests that individuals select regulation partners according to their perceived competence for a specific emotion. We examined whether this extends to regulatory motives, and whether partner–motive pairing is associated with better interaction outcomes, higher psychological well-being, and lower social loneliness.

**Methods:**

In a preregistered 14-day ecological momentary assessment (EMA) study, 106 adults reported on their friend contacts at four semi-random prompts per day (2,796 contact reports). For every contact, participants indicated the primary benefit they had hoped to obtain (one of six affect-improving regulation strategies), the friend involved, how far the contact had helped, and their evaluation of it. Partner-level pairing was quantified with a Specialization Score and, for interaction outcomes, with a leave-one-out residual separating motive-specific pairing from a partner’s overall involvement; network-level pairing with a Network Specialization Index (NSI). Motive-specific pairing was tested against participant-level permutation nulls and with a residualized metric. The strategy a friend actually used was not assessed.

**Results:**

Evidence for motive-specific pairing beyond a partner’s overall involvement was limited and motive-dependent: under the permutation null no motive survived correction for multiple testing, and the residualized metric indicated motive-specific channeling only for problem solving and distraction. An exploratory network-level index standardized against each participant’s own permutation null exceeded chance on average (d = 0.36) but reached significance for only 18 of 104 participants. Neither the leave-one-out predictor nor the network-level indices were associated with perceived emotion regulation effectiveness, interaction evaluation, psychological well-being, or social loneliness. These null findings were robust across alternative metrics, indices, ordinal models, and random-effects structures.

**Conclusions:**

Participants repeatedly paired particular partners with particular motives. Beyond the concentration of contact on a few partners, this pairing was small and was detectable mainly in an aggregate network-level test, and it was not associated with better interaction outcomes or better psychological adjustment. The findings caution against inferring functional specialization from partner-selection data alone and underline the distinction between network indices derived from self-reported contact reports and the subjective experience of support.

**Supplementary Information:**

The online version contains supplementary material available at https://doi.org/10.1186/s40359-026-05767-w.

## Introduction

“Some people go to priests; others to poetry; I to my friends.” With this observation, Virginia Woolf [1] captured a natural human coping behavior: turning to the social network. Empirical work confirms that social networks are important determinants of both mental and physical health, with friends serving as primary sources of emotional support [2]. Social Baseline Theory proposes that humans naturally “outsource” emotional and regulatory load to trusted others [3]. Consequently, individuals should not be viewed as isolated regulators; they frequently and effectively rely on social partners, often shown to be more beneficial than intrapersonal regulation [4–6]. Yet most research has focused on dyadic relationships, leaving the broader friendship network largely unexplored. The present study addresses this gap by examining functional specialization within friendship networks and its implications for interaction evaluation, psychological well-being, and social loneliness. We use functional specialization to denote a network structure in which different partners are reliably associated with different regulatory functions, over and above how often each partner is contacted overall. Its observable footprint in daily-life data is a systematic pairing of partners with regulatory motives, and it is this footprint, rather than functional specialization itself, that the present design can assess. The construct is well established in personal-network research, where it appears under other names. Most network ties provide specialized rather than comprehensive support, so that different members are approached for different kinds of help [7, 8], and people activate problem-specific rather than general discussion networks when a particular need arises [9]. When several relationship-specific networks are mapped in the same community, they are related but not redundant, so that a person who is central in one can be peripheral in another [10]. Within the emotion-regulation literature, Cheung et al. [11] showed that people name distinct partners for distinct emotions, a pattern since replicated in an independent sample [12]. What has not been examined is whether the same differentiation extends to the regulatory motives that structure everyday friend contact.

## Interpersonal emotion regulation

In everyday life, individuals employ various strategies to influence the occurrence, intensity, and quality of their emotional experiences [13–15]. While traditionally studied as an intrapersonal process, emotion regulation (ER) frequently happens within social contexts, through interactions with others: a process known as *interpersonal emotion regulation* (IER) [16–18].

The present study focuses specifically on intrinsic IER, examining how individuals strategically leverage their social network to meet their own emotional needs. This goal-driven nature distinguishes IER from concepts like social buffering, which refers to the reduction of stress simply through the presence of others [3, 19]. Although distinct, these processes are not mutually exclusive; rather, they often interact, as the physical proximity of a close other facilitates the active regulation of emotions, consistent with Social Baseline Theory [20]. Importantly, everyday social contact is not always initiated for regulatory purposes; interactions may be incidental, routine, or serve instrumental and recreational goals. Our operationalization therefore targets the primary benefit individuals hoped to obtain from a given contact (their dominant interaction motive) rather than presupposing an explicit, deliberate regulatory episode. Intrinsic IER is also distinct from social support. Support transactions need not be aimed at changing the recipient’s emotional state, whereas intrinsic IER is defined by that aim, and support can be received without being sought. Evidence that a broader set of support providers is beneficial therefore does not, by itself, establish that a broader set of regulatory partners, or a differentiation of partners by regulatory motive, is beneficial. We treat that literature as motivating rather than as establishing the present predictions.

## Interpersonal emotion regulation strategies

When regulating emotions in the context of social interactions, a wide range of strategies is available. These range from cognitive strategies, such as helping a person see a situation differently, through instrumental and behavioral strategies, such as working through a problem together or offering physical closeness, to attention-oriented strategies, such as drawing attention away from the situation through humor or a shared activity. Following the taxonomy proposed by Niven et al. [21], this study focuses exclusively on affect-improving strategies (those intended to improve the target’s emotional state). This aligns with research consistently suggesting that the majority of interpersonal regulatory efforts aim to reduce negative affect and enhance positive affect rather than worsen it [19, 22]. We selected strategies that represent distinct regulatory mechanisms (cognitive, behavioral, and attention-oriented) to capture the diversity of regulatory needs. Six affect-improving strategies were investigated: problem solving, encouragement, reappraisal, humor, distraction, and touch. Table 1 gives the exact response options by which these motives were presented to participants. Their theoretical background and the literature they derive from are described in Additional file 1, Table S5. Adaptive regulation depends on deploying the appropriate strategy for a given context [23], a principle that extends to the interpersonal domain [24].

## Specialized emotion regulation in friendship networks, well-being and loneliness

Fitzsimons and Shah [25] demonstrated that individuals are more likely to approach others whose characteristics align with their current goals. Consistent with this view, empirical evidence suggests that individuals selectively rely on relationship partners perceived as effective at regulating their specific current emotion [11]. Cheung et al.'s Emotionship model proposes that individuals tend to seek out different friends for different emotional states (e.g., sadness vs. anxiety). Emotion-specific and motive-specific partner selection are related but not equivalent: turning to different friends for different emotions implies turning to different friends for different regulatory motives only to the extent that emotions map onto the kind of help that is sought. Three mechanisms make such a mapping plausible. First, if a partner is approached because they are expected to be effective for a given emotion, and effectiveness is achieved through a particular kind of regulatory response, then partner choice should track the response that is needed rather than the emotion label itself. Second, partners differ in stable attributes that afford some responses more than others, such as humor, practical experience, or physical proximity, so that expectations about what a partner will do become attached to that partner. Third, repeated contacts establish relational scripts in which a specific partner becomes the default for a specific kind of exchange. These mechanisms motivate an extension of the Emotionship framework from emotions to regulatory motives, but they do not guarantee it, because one partner may be perceived as effective across several emotions and motives at once. According to the theory of Perceived Partner Responsiveness, interactions are evaluated most favorably when partners are perceived as understanding and meeting specific needs [26, 27]. The three perspectives contribute distinct predictions. Social Baseline Theory concerns the presence of a trusted other and therefore predicts benefits of social contact largely irrespective of which motive is served [3, 20]. The Emotionship framework concerns whom people approach and predicts differentiation between partners as a function of the emotion to be regulated [11]. Perceived Partner Responsiveness concerns how an interaction is evaluated and predicts benefits when the response received matches the need brought to the interaction [26, 27]. H1 and H3 follow from the Emotionship framework and H2 from Perceived Partner Responsiveness, whereas Social Baseline Theory supplies the alternative account against which both are assessed, because it predicts benefits of contact without requiring motive-specific matching. This aligns with findings by Lopes et al. [28], who suggest that emotion regulation abilities are linked to the quality of social interactions.

Empirical evidence consistently demonstrates that social support is crucial for mental and physical health [29–31]. However, beyond mere support, research indicates that perceived support relies more on the number of available providers than on the depth of any single relationship, suggesting that relying on one person may be insufficient [32, 33]. This literature concerns the provision of support rather than intrinsic emotion regulation, and it shows that having several available providers is associated with better outcomes, not that providers must be differentiated by regulatory motive. Crucially, this appears to be driven by the functional aspects of these ties [33, 34]. In the specific context of emotional support, this implies that the value of a network lies in the variety of strategies it provides. This conceptualization is supported by the *Emotionship* model: Cheung et al. [11] found that individuals derive greater well-being from a diverse portfolio of specialized regulation partners, rather than from a large, undifferentiated network. Just as internal flexibility predicts better psychological functioning [4, 35], a network of specialized contacts provides an interpersonal regulatory repertoire. When support matches the specific regulatory need, this qualitative fit, rather than mere contact, may serve as a protective factor against the sense of isolation that arises when needs remain misunderstood or unmet [36].

Two outcomes are of particular interest in this context. Psychological well-being reflects the degree to which universal human needs, such as competence, relatedness, autonomy, and meaning, are fulfilled, enabling optimal functioning and flourishing [37]. Research suggests that close relationships predict well-being precisely through their role in coping and emotion regulation [16]. Loneliness, in contrast, captures the subjective discrepancy between desired and actual social relationships [38]. This discrepancy often arises when specific emotional needs go unmet [36]. A specialized network addresses this by ensuring that regulatory deficits are matched with effective support, minimizing unmet needs and ensuring that social interactions are functionally satisfying, rather than just frequent. Two features of this rationale require explicit statement. First, differentiation is predicted to add to, rather than merely reflect, the availability of partners. A network in which several partners can serve the same function already provides redundancy; a network in which partners are differentiated additionally provides coverage, that is, a fitting partner for a wider range of needs. Only the latter predicts an incremental association once network size, the number of close friends, and general support availability are held constant, which is why these variables enter the models as covariates. Second, the two outcomes are reached by different routes. Psychological well-being is expected to benefit cumulatively, because a larger proportion of regulatory episodes ends in a fitting response. Social loneliness, by contrast, refers to a perceived deficit in one’s wider social circle rather than in a single close attachment [38, 39], so a differentiated network should reduce it because fewer needs are left unmet across the circle as a whole. We therefore specify social loneliness, rather than emotional or overall loneliness, as the outcome of H3b. Where the evidence cited above does not distinguish loneliness subtypes, we treat it as indirect support for this prediction.

Conceptually, network-level differentiation is the structural aggregate of the interaction-level matching processes examined in this study: if individuals repeatedly turn to motive-specific partners and benefit from well-matched interactions, then networks offering a wider repertoire of specialized partners should, cumulatively, protect well-being and prevent the experience of unmet regulatory needs that fosters loneliness. Testing all three levels within one design allows us to locate where in this chain the presumed benefits of specialization emerge or break down.

## The present study

The present study examines functional specialization within friendship networks. While Cheung et al. [11] linked specific friends to specific emotions, we ask whether an analogous pairing is observable at the level of regulatory motives, that is, of the benefit a person hopes to obtain from a contact. Because the design records the motive a participant reported and the friend they contacted, but not the response that friend actually gave, the hypotheses below concern partner–motive pairing and its correlates rather than the provision or the effectiveness of particular strategies. Based on the theoretical framework outlined above, the following hypotheses are proposed:

### H1: Motive-specific partner selection

Participants will exhibit preferential selection within their networks, contacting a specific “go-to” friend for a given regulatory motive more often than expected by chance. Specifically, we predict that the maximum Specialization Score per motive will exceed chance levels. H1 concerns the strength of the link between one motive and one partner. It does not concern whether different motives are served by different partners, which is a network-level property addressed in H3. Chance is defined by a participant-level permutation null that preserves each friend’s overall involvement rather than by the preregistered 1/k benchmark (see Statistical Analyses).

### H2: Interaction outcomes

We hypothesize that the alignment between a network member’s motive-specific pairing and the motive reported for the current contact will predict better interaction outcomes. Specifically, contacting a friend who is strongly paired with the motive reported for that contact will be associated with higher perceived ER effectiveness, that is, with participants reporting that the contact helped them achieve their desired emotional state (H2a), and with a better evaluation of the interaction (H2b).

### H3: Outcomes of specialized friendship networks

Extending the concept of regulatory flexibility to the social level, we hypothesize that individuals benefit from having a diverse social network, which consists of various friendships specialized for different emotion regulation strategies. Specifically, we predict that higher scores on the Network Specialization Index (NSI) will be positively associated with psychological well-being (H3a) but negatively associated with social loneliness (H3b). As set out in the Measures and revisited in the Discussion, the NSI summarizes how strongly each motive is concentrated on its most frequently contacted partner. It does not establish that different motives are served by different partners, and H3 is interpreted accordingly.

## Methods

### Participants

An a-priori power analysis was conducted using the “Power Curves for Multi-level Studies” tool provided by the Kleiman Lab at Rutgers [40]. Results indicated that a minimum sample size of 100 participants was required to identify a medium effect size (Cohen’s *d* = 0.50) for within-person effects with acceptable statistical power (1-β = 0.80). These estimates assumed an average completion rate of at least 75% of the time-based EMA prompts and estimated intraclass correlation coefficients (ICCs) of approximately 0.4 for positive and negative affect [40, 41]. Because this power analysis targeted within-person effects, we additionally conducted a sensitivity analysis for the between-person analyses (Hypothesis 3): with *N* = 106, α = 0.05, power = 0.80, and five covariates, the minimum detectable increment for the NSI corresponds to *f*² = 0.079 (ΔR² ≈ 7.3%). The between-person analyses were thus powered to detect medium-sized increments only, which should be kept in mind when interpreting their results. To account for potential dropouts or data exclusion, 124 participants were recruited using the SONA Participant Management System of the Institute of Psychology, University of Würzburg. Participants received either monetary compensation of up to €18, contingent on the number of EMA prompts completed, or, for Bachelor’s students, research participation credits.

Study requirements included the minimum age of 18 years, sufficient understanding of the German language and residing in the Central European Time zone (CET) during data collection. Participants who reported having been diagnosed with, or treated for, a mental health condition within the past 12 months were excluded prior to data collection. This decision was made because acute mental health conditions are associated with altered patterns of social interaction and support seeking, which would confound the network processes under study. It also avoided subjecting potentially vulnerable individuals to a two-week self-monitoring protocol without clinical supervision. Following data collection, participants were excluded if they completed less than 50% of the prompts (< 28 prompts), failed to complete the baseline or post-EMA surveys, or reported no friend interactions during the study period (preregistered exclusion criteria: https://doi.org/10.17605/OSF.IO/MBJRPhttps://osf.io/mbjrp/overview?view_only=e41c0c16ef3e4fae88ed0cfef5540085). Applying these criteria in sequence, 15 participants were excluded because they had completed fewer than 28 valid prompts and two further participants because they had not completed the final survey. No participant was excluded solely for reporting no friend contact, because the one participant without any reported friend contact had already been excluded for low compliance. One further participant was excluded due to incoherent responses (see “Data preparation and cleaning”). Of the 124 recruited participants, 106 were therefore retained. The final sample consisted of *n* = 106 participants (80 female, 24 male, 1 non-binary, 1 prefer not to say; mean age = 24.72 years, SD = 7.97). The sample consisted largely of university students or high-school graduates: 73.6% held a university entrance qualification (Abitur) as their highest educational level and 15.1% held an academic degree (Bachelor’s or Master’s). Regarding relationship status, 59.4% reported being in a romantic relationship, and the majority (93.4%) had no children. Person-level descriptive statistics for the central study variables are reported in Table 2.

### Procedure

The study was conducted between June 6, 2025, and July 18, 2025, and consisted of three distinct phases: a baseline assessment, a 14-day EMA phase, and a final survey. Participants started the EMA phase themselves after completing the baseline survey. Once the EMA phase had started, the schedule was highly uniform: the median interval between a participant’s first and last prompt was 13.33 days (range 13.27–13.39). The final survey followed the last EMA prompt immediately (median delay 1.2 h, interquartile range 1.0–1.9 h), and 103 of the 106 participants completed it within 24 h.

#### Baseline assessment

The baseline survey was administered via SoSci Survey [42]. After providing informed consent and agreeing to the data privacy statement, participants reported their demographic information. In line with study requirements, the baseline survey also included self-reported exclusion criteria regarding current mental health diagnoses. Upon completing the baseline assessment, participants received detailed study instructions, a personal identification code to link their data, and a guide for downloading and configuring the m-Path smartphone application [43]. This allowed participants to create a user account and initiate the EMA phase at a time of their choice.

#### EMA-phase

Over 14 consecutive days, participants received four prompts per day via the m-Path app. Prompts were sent semi-randomly within four fixed 120-minute time blocks (09:00 a.m.–11:00 a.m., 12:00 p.m.–02:00 p.m., 03:00 p.m.–05:00 p.m., and 06:00 p.m.–08:00 p.m.). A reminder notification was sent 30 min after the initial prompt, and questionnaires expired after 60 min to prevent overlapping prompts and ensure an interval of at least one hour between assessments. Each prompt began by asking whether participants had had contact with a friend since the previous assessment (“Have you had contact with a friend since the last survey?”; response options: in person; virtually via call or text message; no contact). Interactions with persons participants did not consider friends were thus not eligible for report. If contact was reported, participants named the friend with whom they had had the most contact since the previous prompt (by initials) and answered all subsequent questions with respect to the contact with this friend. Consequently, one friend contact was assessed per prompt; multiple contacts with the same friend within one interval were aggregated into a single report, and additional friends contacted in the same interval were not assessed at that prompt. When participants nevertheless listed several friends, all named friends were retained and weighted accordingly (see Measures). Branching logic was used to ensure similar completion time (2–4 min) regardless of whether a social interaction was reported, thereby reducing the incentive to omit interactions to shorten survey completion. Participants then indicated, in a single-choice format, which of six benefits they had most hoped to obtain from the contact (“What did you hope most to get out of this contact?”). The item did not include an opt-out option (see Limitations). Because the prompt did not first establish that a regulatory need had been present, offered no opt-out, and did not ask what the friend had actually done, this variable records the benefit a participant hoped to obtain from a contact. It does not record a verified regulatory episode, and it does not record a strategy enacted by the friend. If no interaction was reported, participants were routed to an alternative pathway: instead of questions about the interaction, they were asked to describe their current activity and whether it helped them regulate their emotions. However, as the present study focuses specifically on interpersonal emotion regulation, the data collected from the pathway without social interaction were not included in the subsequent analyses. To ensure response quality, two attention checks were embedded in each prompt. Specifically, Item 4 instructed participants to “Please select ‘a little’ if you are currently answering the questions attentively”, while Item 9 instructed them to “Please select ‘I completely agree’ if you are currently answering the questions attentively”. Prompts were excluded from the analysis if participants failed both attention checks, which applied to 5 of the 5,049 prompts completed in the final sample, or if the reported contact partner was given as a collective term such as “friends” or “many people” rather than as an identifiable person, which applied to 2 further prompts. The remaining 5,042 prompts entered the analyses. The complete item order and the German wording are provided in the OSF Repository.

#### Follow-up assessment

Immediately following the final EMA prompt, participants received the final questionnaire via the m-Path app, with multiple reminders sent to encourage completion. The post-assessment included measures of psychological well-being and loneliness (see below), as well as a checklist of stressors experienced during the study period.

### Measures

#### Demographics and social network

In the baseline questionnaire, participants provided demographic details (age, gender, income, subjective social status, relationship, and parental status). Additionally, they reported the size of their social network by indicating the number of individuals they considered close friends. Note that the EMA network (all friends named during the 14 days) is conceptually broader than these baseline nominations of close friendships; “friend” was participant-defined and the relationship type of EMA partners was not independently verified.

#### Reported regulatory motive

One item assessed the primary benefit participants had hoped to obtain from the reported contact, corresponding to one of six affect-improving IER strategies. Throughout, we refer to this variable as the reported regulatory motive, and we use the six strategy names only as labels for its response categories. Participants selected from six distinct strategies: problem solving, encouragement, reappraisal, humor, distraction, and touch. Response options were presented as short descriptive sentences rather than as technical labels. The six options are reported in Table 1. Participants were restricted to selecting a single option representing their primary motive; the item did not include a “none of these” option, so that contacts without any affect-improving motive could not be identified as such.

**Table 1:** Response Options of the Item Assessing the Reported Regulatory Motive

Regulatory motive	Response option presented to participants
Problem solving	“That they listen to me, give advice, and help me actively address a problem or issue.”
Encouragement	“That they encourage me, highlight my strengths, and believe in me.”
Reappraisal	“That they support me and help me see the situation from a different perspective.”
Humor	“That they make me laugh and cheer me up.”
Distraction	“That they distract me and give me a pleasant break.”
Touch	“That they comfort me with a hug and give me closeness.”

Note. Participants answered the single-choice item “What did you hope most to get out of this contact?” at every prompt at which a friend contact was reported. The options were presented in German and without the labels given in the left-hand column. The English translations are shown here and the German originals are available in the OSF repository. The theoretical background of the six motives is described in Additional file 1, Table S5.

#### Perceived ER effectiveness

Participants’ perception of whether the contact had helped them achieve their desired emotional state was assessed with a single item (“To what extent did this social interaction help you achieve your desired emotional state?”). Responses were given on a 7-point scale ranging from 1 (Not at all) to 7 (Completely). We refer to this variable as perceived ER effectiveness. The variable is a single self-report of perceived goal attainment: it does not index the partner’s regulatory skill, the strategy the partner used, or an observed change in affect.

#### Interaction evaluation

Participants evaluated the quality of the contact on four dimensions using 7-point scales: *Closeness* (“How close did you feel to the person?”), *Helpfulness (“Was the contact helpful?”)*,* Pleasantness (“Was the contact pleasant?”)*,* and Satisfaction (“Are you satisfied with the contact?”)*. A composite Interaction Evaluation Index was computed as the mean of these four items, with higher scores indicating higher interaction evaluation. In the present sample, internal consistency of the four items across all 2,796 observations was high (Cronbach’s α = 0.90). Because the composite is measured repeatedly within persons, we additionally decomposed its reliability with a multilevel generalizability analysis (multilevel.reliability in the R package *psych*). The composite reliably captured within-person, occasion-to-occasion variation (R*cn* = 0.85), and person means aggregated across all of a participant’s occasions were highly reliable (R*kRn* > 0.99). The latter coefficient is high mainly because each participant contributed many occasions and must not be read as the reliability of a single momentary rating. All generalizability coefficients and the underlying variance components are reported in Additional file 1, Table S10.2.

#### Contextual variables

To account for situational context, participants characterized each interaction by its type (physical/in-person, virtual/video or phone, or text-based) and the concurrent activity (e.g. sports, studying, working, household chores, leisure activities). For the main analyses, phone/video and text-based contacts were collapsed into a single “virtual” category, consistent with the preregistered physical-versus-virtual contrast; a sensitivity analysis using the full three-level modality factor (in-person, synchronous virtual, text-based) is reported in Additional file 1 (SectionS11).

#### Network specialization indices

To quantify partner–motive pairing, two indices were derived from the EMA data. For every interaction where a regulatory motive was reported, participants explicitly identified the specific network member involved. Based on this data, a Specialization Score was calculated to quantify the extent to which the contacts reported for a specific motive were concentrated on a specific friend. The Score was calculated for every Friend × Strategy combination. To account for interactions involving multiple friends, the preregistered formula was adjusted to apply proportional weighting: if *n* friends were present during a single interaction, each friend received a weight of 1/*n* for that entry. Scores range from 0 to 1, with higher values indicating that Friend i was contacted more consistently when the participant reported Motive j. The score is calculated as:

$$\begin{array}{lll}{\mathrm{Specialization}} \; {\mathrm{Score}}_{\mathrm{i,j}} = \\ \frac{{\Sigma\:}\:\mathrm{W}\mathrm{e}\mathrm{i}\mathrm{g}\mathrm{h}\mathrm{t}\:\mathrm{o}\mathrm{f}\:\mathrm{f}\mathrm{r}\mathrm{i}\mathrm{e}\mathrm{n}\mathrm{d}\:i\:\mathrm{i}\mathrm{n}\:\mathrm{e}\mathrm{n}\mathrm{t}\mathrm{r}\mathrm{i}\mathrm{e}\mathrm{s}\:\mathrm{w}\mathrm{h}\mathrm{e}\mathrm{r}\mathrm{e}\:\mathrm{S}\mathrm{t}\mathrm{r}\mathrm{a}\mathrm{t}\mathrm{e}\mathrm{g}\mathrm{y}\:j\:\mathrm{w}\mathrm{a}\mathrm{s}\:\mathrm{n}\mathrm{e}\mathrm{e}\mathrm{d}\mathrm{e}\mathrm{d}}{\mathrm{T}\mathrm{o}\mathrm{t}\mathrm{a}\mathrm{l}\:\mathrm{e}\mathrm{n}\mathrm{t}\mathrm{r}\mathrm{i}\mathrm{e}\mathrm{s}\:\mathrm{w}\mathrm{h}\mathrm{e}\mathrm{r}\mathrm{e}\:\mathrm{s}\mathrm{t}\mathrm{r}\mathrm{a}\mathrm{t}\mathrm{e}\mathrm{g}\mathrm{y}\:j\:\mathrm{w}\mathrm{a}\mathrm{s}\:\mathrm{n}\mathrm{e}\mathrm{e}\mathrm{d}\mathrm{e}\mathrm{d}}\end{array}$$

For the interaction-level analyses (H2), the Specialization Score of the named friend was used; when several friends were listed for one contact, the score of the first-named friend was used, and a sensitivity analysis using the weighted mean score across all listed friends is reported in Additional file 1.

Because the Specialization Score is a conditional proportion, it can conflate motive-specific pairing with a friend’s overall involvement across all interactions (tie strength, availability, contact frequency). To address this, we additionally computed a residualized specialization metric: the standardized Pearson residual of the participant-level friend × strategy contingency table, where expected values derive from the table margins under independence. Positive values indicate that a friend was contacted for a given motive more often than expected from that friend’s overall contact frequency. For the interaction-level analyses (H2), the same logic was applied report by report. For every contact report, the participant’s friend × motive table was recomputed without that report, and the standardized Pearson residual of the cell formed by the first-named friend and the motive reported for that contact served as the focal predictor (leave-one-out partner–motive residual). This predictor therefore neither contains the observation whose outcome it predicts nor confounds motive-specific pairing with the partner’s overall involvement. It could be computed for 2,430 of the 2,796 contact reports (86.9%). In the remaining reports the focal contact was the participant’s only report of that motive or the only contact with that partner, so that the leave-one-out table held no information for the corresponding cell. Retained and non-computable reports differed little in the outcomes (perceived ER effectiveness M = 5.58 vs. 5.54; interaction evaluation M = 5.73 vs. 5.69). As intended, the new predictor was moderately correlated with the original Specialization Score (*r* = .48) but, unlike that score (*r* = .73), was essentially uncorrelated with the partner’s overall share of a participant’s contacts (*r* = − .07).

Second, we derived the *Network Specialization Index* (NSI*)* to summarize, across motives, how strongly each motive was concentrated on its most frequently contacted partner. To this end, for each of the six strategies, the friend with the highest Specialization Score was identified as that motive’s primary partner. The index was then calculated as the mean of these maximum scores across all strategies used by the participant. A higher NSI indicates that, on average, each motive was concentrated on a single partner. Because the same partner can hold the maximum score for several motives, a high NSI does not imply that different motives are served by different partners.

$$\begin{array}{lll}{\mathrm{NSI}}_{\mathrm{Participant}}= \\ \:\frac{\left({Max\:Specialization\:Score}_{ER-Strategy\:1}+\:{Max\:Specialization\:Score}_{ER-Strategy\:2}+\:\dots\:\:+\:{Max\:Specialization\:Score}_{ER-Strategy\:n}\right)\:}{Total\:number\:of\:used\:strategies}\end{array}$$

Because the NSI averages maximum scores, it cannot distinguish a differentiated network (different specialists per strategy) from reliance on a single friend across all strategies. We therefore additionally computed the Functional Differentiation Ratio (FDR), defined as the number of distinct primary partners divided by the number of motives reported, and the effective number of partners, exp(H), where H is the Shannon entropy of a participant’s weighted contact distribution across friends. Higher values on both indices indicate that contacts were spread over more partners. In what follows we therefore describe the NSI as a concentration index and the FDR and the effective number of partners as diversity indices. None of the three quantifies the strength of the association between partner identity and regulatory motive at the network level. We therefore added a network-level partner–motive association index. For each participant, the χ² statistic of the weighted friend × motive table was compared with the distribution of the same statistic across 5,000 permutations of the motive labels over that participant’s contact reports. The resulting z-score expresses how much more partner–motive structure a participant’s network shows than is expected from that participant’s own number of reports per motive and each friend’s overall involvement. It is therefore adjusted for network size and for contact frequency. This index was not preregistered, and analyses using it are reported as exploratory. In the present sample, the NSI and the FDR were only weakly correlated (*r* = − .30), confirming that concentration and differentiation capture distinct constructs.

#### Psychological well-being

Psychological well-being was measured with the German adaptation of the Flourishing Scale (FS-D) [44, 45]. The questionnaire reflects psychological well-being in the sense of optimal human functioning and consists of 8 items with a 1 (Strongly disagree) to 7 (Strongly agree) response scale (e.g., “I lead a purposeful and meaningful life”). The items were adapted asking specifically about the last two weeks. To calculate the individual result, the values of each answer are added, resulting in a possible range of scores from 8 to 56, where a high score indicates high psychological well-being and flourishing. The scale demonstrated good reliability (Cronbach’s α = 0.79) and acceptable validity, with correlations with other measures ranging from 0.43 to 0.69 [45]. In the present sample, internal consistency of the adapted scale was high (Cronbach’s α = 0.90, McDonald’s ω = 0.90).

#### Loneliness

Loneliness was assessed using the Social Loneliness subscale of the Social and Emotional Loneliness Scale for Adults (SELSA) [39]. This subscale consists of 14 items rated on a 7-point Likert scale ranging from 1 (Strongly disagree) to 7 (Strongly agree). An example item is “I don’t have a friend(s) who shares my views, but I wish I did”. For the present study, items were adapted to explicitly refer to the past two weeks to match the EMA observation period. To ensure linguistic and conceptual equivalence, the original English items underwent a multi-stage translation and validation procedure. First, two independent bilingual researchers translated all items into German. The translations were consolidated into a consensus version, which was subsequently back-translated using translation tools (DeepL and ChatGPT 5.0). Discrepancies between the original and back-translated items were reviewed and resolved to maximize accuracy. For data analysis, a total score was computed by summing all items, resulting in a range of 14 to 98, with higher scores indicating greater social loneliness. The scale demonstrates good internal consistency (Cronbach’s α = 0.91) and evidence of discriminant validity, with the social subscale correlating more strongly with social loneliness measures than with emotional loneliness [39]. In the present sample, the adapted German version showed high internal consistency (Cronbach’s α = 0.92, McDonald’s ω = 0.93). Internal consistencies are summarized in the Additional file 1, Section S10.

#### Stressors

To account for external influences on social interactions and well-being, presence of acute stressors was assessed. Participants indicated (*yes/no*) whether they had experienced any events that affected them more than usual during the study period. Examples were provided to guide responding (e.g. recent separation from a partner, bereavement, exposure to violence, unexpected job loss, etc.). Participants who responded affirmatively were given the option to describe the event in an open-text format.

### Statistical analyses

All data management and statistical analyses were conducted using R (Version 4.5.1) within the RStudio environment (Version 2025.09.1). The primary packages used were *psych* for descriptive statistics, *lme4* for multilevel modeling, and *ggplot2* for data visualization [46–48]. Statistical workflows were supported by AI tools to streamline coding and data analyses (Claude Sonnet 4.5 and ChatGPT 5.0).

#### Data preparation and cleaning

Prior to analysis, data were screened and prepared for statistical processing. The full data-cleaning procedure is documented in the R script provided in the OSF Repository. Regarding demographic variables, the categories “Diverse” and “No answer” in the gender variable were combined into a single category labeled “Other” due to extremely small group sizes (*n* = 1 each). For transparency, we report the standardization process applied to participants’ free-text friend nominations. To resolve formatting inconsistencies in the raw entries (e.g., “M.F.,” “MF,” “M F,” “mf”), a two-step cleaning procedure was applied. First, an automated string-processing script was used to standardize the format: all text was converted to uppercase, punctuation (periods, leading/trailing quotation marks) was removed, and newline characters were replaced with commas to delineate multiple friends. Second, the dataset was exported for manual review to resolve remaining irregularities. During this phase, inconsistencies such as the use of conjunctions (e.g., ‘and’) or incorrect numbering were manually corrected. Entries that could not be plausibly resolved were coded as invalid. Following the data cleaning, general exclusion criteria were applied to the full sample. From the remaining valid cases, one participant was excluded due to systematic inconsistency in their naming conventions (e.g., alternating between initials, full names, and role descriptions such as ‘mother’), which made it impossible to reliably identify unique network members.

#### Data analytic strategy

First, descriptive analyses were conducted to characterize the sample at the person level and to summarize EMA-level data, including the frequency of social interactions and the utilization rates of the different emotion regulation (ER) strategies.

Hypothesis 1 was preregistered to be tested with one-tailed paired-samples *t*-tests comparing each participant’s maximum Specialization Score per strategy against an individual chance probability of 1/k (k = number of friends in the EMA network). However, the maximum of k observed proportions systematically exceeds 1/k even under random allocation, and the 1/k benchmark ignores that contact is typically concentrated on few partners. As the primary test of H1, we therefore used participant-level permutation tests: within each participant, strategy labels were permuted across that participant’s reported contacts (5,000 iterations), which preserves both the participant’s number of occasions per strategy and each friend’s overall involvement (i.e., both margins of the friend × strategy table). For each strategy, the observed maximum Specialization Score was compared against the resulting null distribution, yielding participant-specific empirical *p*-values. At the group level, the observed mean maximum score was compared against the permutation distribution of the mean. Strategies with fewer than three occasions for a given participant were excluded from this test, and Bonferroni-Holm correction was applied across the six strategy-level tests. The same permutation framework was applied to the residualized specialization metric. The preregistered t-tests (and non-parametric Wilcoxon tests) are retained in the Additional file 1 Tables S1a and S1b for transparency. Neither the permutation framework nor the residualized metric was preregistered; both are reported throughout as non-preregistered analyses. For Hypothesis 2, two linear mixed-effects models were estimated, predicting perceived ER effectiveness (H2a) and interaction evaluation (H2b) at Level 1. The primary specification included random intercepts for participants and for partners nested within participants. Because each partner belongs to exactly one participant, these random effects are nested rather than crossed. This structure was chosen on substantive grounds: the 2,796 observations are repeated measures on 802 identifiable, participant-specific partners (M = 7.57 partners per participant; M = 3.49 observations per partner), and the focal predictor is itself a partner-level quantity, so omitting the partner level would leave partner-induced dependency in the residual term and bias the test of the focal predictor. The primary H2 analyses are restricted to the 2,430 contact reports for which the leave-one-out predictor could be computed (104 participants, 508 partners, M = 4.78 reports per partner). In this analysis sample, adding the partner intercept improved model fit substantially for both outcomes (H2a: χ²(1) = 73.05; H2b: χ²(1) = 133.18; both *p* < .001). A random slope for the focal predictor did not improve model fit for either outcome (perceived ER effectiveness: χ²(2) = 2.41, *p* = .300; interaction evaluation: χ²(2) = 2.05, *p* = .358), and neither random-slope model produced a singular fit or a convergence warning. The random-intercept structure is therefore retained on statistical grounds rather than for comparability. For the original Specialization Score, a random slope had improved fit for perceived ER effectiveness (χ²(2) = 6.50, *p* = .039) but not for interaction evaluation (χ²(2) = 4.43, *p* = .109), without changing the fixed effect. Those models are retained in Additional file 1. The specification with a participant random intercept only, is retained as a sensitivity analysis in Additional file 1 (Tables S9.1 and S9.2). Modeling proceeded in two steps: The baseline model included age and gender as Level-2 fixed covariates, and Interaction Type (physical vs. virtual) and IER Strategy as Level-1 fixed covariates. The full model added the leave-one-out partner–motive residual of the contacted friend as a Level-1 continuous fixed predictor (see Measures). This specification was chosen as the primary test of H2 because it avoids circularity between predictor and outcome and because it isolates motive-specific pairing from the partner’s general involvement. Three further versions of the predictor are reported as sensitivity analyses: the original Specialization Score, a leave-one-out version of that score, and a lagged version based only on contact reports preceding the focal one. Continuous predictors were centered to minimize multicollinearity and facilitate interpretation: age was grand-mean centered, and the focal predictor was person-mean centered to examine within-person effects. Model assumptions were evaluated by inspecting residuals for normality (Q–Q plots), homoscedasticity, and linearity. Intraclass correlation coefficients (ICCs) were calculated to quantify the proportion of variance attributable to the participant level and to the partner level. Models were estimated using Maximum Likelihood (ML) for model comparisons and Restricted Maximum Likelihood (REML) for final parameter estimation. Fixed effects were interpreted at a significance level of *p* < .05. To examine whether network size confounded the H2 results, a sensitivity analysis was conducted adding EMA network size as an additional covariate to the baseline models. Additional sensitivity analyses (a) re-estimated the model for perceived ER effectiveness as a cumulative link mixed model to accommodate the ordinal, negatively skewed outcome, (b) used the three-level modality factor (in-person, synchronous virtual, text-based) instead of the binary contrast, and (c) excluded touch as a sparse category; distributional characteristics of both outcomes, including the proportion of ratings at the scale maximum, are reported in the Additional file 1 (Table S9.3).

Finally, Hypothesis 3 was tested using two hierarchical linear regressions predicting psychological well-being (H3a) and loneliness (H3b) using the Network Specialization Index (NSI). Predictors were entered in two steps: the baseline model included age, gender, number of close friends, and current stressors. The full model additionally included NSI to examine its unique contribution beyond the covariates. Sensitivity models additionally adjusted for EMA network size and the number of observations per strategy, both of which can mechanically influence specialization scores, and parallel models replaced the NSI with the FDR, with the effective number of partners and, as an exploratory analysis, with the network-level partner–motive association index. All regression coefficients are reported with 95% confidence intervals. Assumptions of linearity and homoscedasticity were assessed using residuals-versus-fitted plots. Normality of residuals was checked via Q–Q plots and the Shapiro–Wilk test, and potential outliers were identified using Cook’s distance.

Apart from the Bonferroni–Holm correction applied to Hypothesis 1, no further alpha adjustments were made. As the hypotheses were specified a priori and address distinct, theory-driven research questions, additional corrections were considered overly conservative and inappropriate for this confirmatory study context.

## Results

### Descriptive analyses

The final sample consisted of 106 participants, whose demographic characteristics are reported in the Participants section. Table 2 presents the descriptive statistics for the central study variables at the person level and their correlations.

Compliance during the EMA phase was high: participants completed an average of 47.63 of the 56 scheduled prompts, that is 85.1% of all scheduled prompts (5,049 of 5,936). Seven completed prompts (0.1%) were removed, five because both attention checks had been failed and two because the reported contact partner was a collective term rather than an identifiable person, leaving 5,042 valid daily prompts. In total, 55.5% of the valid prompts involved friend contact, corresponding to 2,796 contact reports and to an average of 26.38 contact reports per participant (SD = 10.00). These contacts occurred primarily in person (70.5%) rather than virtually (29.5%). Of the virtual contacts, approximately two thirds were text-based and one third occurred via phone or video call. Among the regulation strategies, distraction (34.1%) and humor (27.4%) were the most frequently reported, followed by problem solving (17.5%), reappraisal (9.1%), and encouragement (8.4%), whereas touch was rare (3.6%). Participants consistently evaluated these contacts favorably, with a mean score of 5.73 (SD = 1.03) on the Interaction Evaluation Index. The average EMA network comprised 8.90 unique partners and thus exceeded the number of close friendships nominated at baseline (*M* = 5.67), consistent with the EMA item capturing the broader friendship circle rather than close friends only. This figure counts every friend a participant named, including those named in the 16.5% of contact reports that listed more than one friend (M = 1.29 friends per report). The mixed models assign each contact report to the first-named friend and therefore contain fewer partners: 802 participant-specific partners, or 7.57 per participant. Because the EMA instruction restricted reports to friends, partners outside the friendship network were not eligible for report.

**Table 2:** Descriptive Statistics and Correlations for Person-level Variables (*N* = 106)

Variable	M (SD)	1	2	3	4	5	6	7	8
1. Age	24.72 (7.97)	-							
2. Subj. Social Status	5.01 (1.55)	− 0.01	-						
3. Close Friends	5.67 (2.93)	0.04	− 0.32^***^	-					
4. Network Size (EMA)	8.90 (4.14)	− 0.04	− 0.12	0.37^***^	-				
5. Total Interactions	26.38 (10.00)	− 0.32^***^	− 0.08	0.31^**^	0.44^***^	-			
6. Mean Interaction Evaluation	5.73 (0.53)	0.15	− 0.11	0.19	0.18	0.00	-		
7. NSI	0.58 (0.14)	0.06	0.01	− 0.21^*^	− 0.52^***^	− 0.18	0.04	-	
8. Loneliness (SELSA)	27.89 (11.76)	0.14	0.21*	− 0.30^**^	− 0.29^**^	− 0.20^*^	− 0.51^***^	0.14	-

Notes. Network Size (EMA) refers to the total count of unique interaction partners reported across the 14-day period. Close friends refers to the baseline nominations. Subj. Social Status = MacArthur Scale of Subjective Social Status (Range 1–10, with higher values indicating higher subjective social status). Potential range for NSI is 0–1. Potential range for SELSA Social Loneliness is 14–98.

* p < .05. ** p < .01. *** p < .001

### Hypothesis tests

#### Hypothesis 1: Motive-specific pairi

The preregistered t-tests against the 1/k benchmark indicated that the maximum Specialization Score exceeded chance for all six motives (all Holm-corrected p < .001, Cohen’s d = 1.67–2.69; full results, including non-parametric Wilcoxon tests, are provided in Tables S1a and S1b, Additional file 1). However, as outlined in Statistical Analyses, this benchmark is inflated by an order-statistics artifact and does not account for the concentration of contact on few partners. The participant-level permutation tests (Table 3) yielded a markedly different picture: at the group level, the observed maximum Specialization Scores did not exceed the permutation null for any motive after Holm correction: nominally significant effects emerged for touch (z = 2.43, perm = 0.010, Holm-corrected p = .061; note, however, that only n = 10 participants contributed ≥ 3 touch episodes), encouragement (z = 2.25, perm = 0.015, Holm p = .074), and reappraisal (z = 2.11, perm = 0.019, Holm p = .077), whereas problem solving, humor, and distraction clearly did not exceed the null (perm = 0.074–0.351). At the participant level, the proportion of individuals whose observed score exceeded their individual permutation null (p < .05) ranged from 4.6% (humor) to 14.3% (encouragement). The residualized specialization metric, which indexes whether a friend’s involvement in a motive exceeds their overall involvement, showed a complementary pattern: significant motive-specific channeling for problem solving (z = 3.29, perm < 0.001, Holm p = .005) and distraction (z = 2.44, perm = 0.008, Holm p = .039), but not for the remaining motives (Additional file 1, Table S7.1). As an exploratory addition, the network-level partner–motive association index tests the same question per participant as an omnibus comparison rather than motive by motive. The index could be computed for 104 of the 106 participants. For the remaining two, every permutation of the motive labels produced the same χ², so the null distribution had zero variance and no standardized score was defined. Averaged over these 104 participants, the observed partner × motive association exceeded the permutation null (M z = 0.49, 95% CI [0.23, 0.75], SD = 1.35; t(103) = 3.70, p < .001, d = 0.36), and 18 of them (17.3%) exceeded their individual null at p < .05. Taken together, the apparent “go-to” pattern under the 1/k benchmark largely reflects the general concentration of contact on few partners. Evidence for motive-specific pairing beyond overall involvement is limited and motive-dependent at the level of single motives, and the aggregate network-level test indicates that a small association is nevertheless present on average.

**Table 3:** Participant-level Permutation Tests for H1 (5,000 Iterations; ≥ 3 Occasions per Strategy)

Strategy	*n*	M(obs)	M(null)	z	*p* perm	*p* (Holm)	% *p* < .05
Problem Solving	70	0.486	0.462	1.48	0.074	0.222	5.7
Encouragement	35	0.544	0.486	2.25	0.015	0.074	14.3
Reappraisal	42	0.524	0.472	2.11	0.019	0.077	7.1
Humor	87	0.460	0.455	0.36	0.351	0.523	4.6
Distraction	92	0.439	0.432	0.63	0.262	0.523	5.4
Touch	10	0.612	0.482	2.43	0.010	0.061	10.0

*Note.* Within each participant, strategy labels were permuted across reported contacts, preserving strategy occasion counts and each friend’s overall involvement. M(obs)/M(null) = observed/permutation-null group mean of the maximum Specialization Score; z = (observed mean − null mean)/SD of null means; p perm = proportion of permutation means ≥ observed mean; % *p* < .05 = share of participants exceeding their individual permutation null. p-values Holm-corrected across the six strategies

#### Hypothesis 2: Interaction outcomes with specialized friend

To test Hypotheses 2a and 2b, two series of linear mixed models were estimated. All models reported below use the primary specification, that is the leave-one-out partner–motive residual as the focal predictor and random intercepts for participants and for partners nested within participants (2,430 contact reports from 104 participants and 508 participant-specific partners). Results for H2b are presented in Table 4; the corresponding models for H2a are reported in Table S2 (Additional file 1). Assumption checks for both models confirmed satisfactory linearity, homoscedasticity, and acceptable normality of residuals and random effects. Multicollinearity was not problematic (VIF < 5), and no influential outliers were identified. Although Q-Q plots indicated a negative skew typical of high-valence rating data, the large sample size (*n* = 2,430) supports the robustness of the estimates. Given this skew and the possibility of ceiling effects (13.1% of evaluation ratings and 23.0% of effectiveness ratings across all 2,796 contact reports were at the scale maximum), a cumulative link mixed model for perceived ER effectiveness was estimated as a sensitivity analysis and confirmed the conclusion (Additional file 1, Table S9.1).

**Table 4:** Results of Hierarchical Linear Mixed Models Predicting Interaction Evaluation (*N* = 2,430 contact reports from 104 participants and 508 participant-specific partners)

Predictor	Null Model	Baseline Model	Full Model
Intercept	5.74 (0.05) ***	5.89 (0.07) ***	5.89 (0.07) ***
**Level-2 Covariates**			
Age		0.02 (0.01) **	0.02 (0.01) **
Gender: Male		−0.25 (0.13)	−0.25 (0.13)
Gender: Other		−0.29 (0.38)	−0.29 (0.38)
**Level-1 Covariates**			
Interaction type: Virtual		−0.53 (0.05) ***	−0.53 (0.05) ***
Strategy: Encouragement		0.15 (0.08)	0.15 (0.08)
Strategy: Reappraisal		−0.05 (0.07)	−0.05 (0.07)
Strategy: Humor		0.21 (0.06) ***	0.21 (0.06) ***
Strategy: Distraction		−0.03 (0.06)	−0.03 (0.06)
Strategy: Touch		−0.20 (0.12)	−0.19 (0.12)
**Main Predictor**			
Partner–motive residual (leave-one-out)			−0.02 (0.02)
**Random Effects**			
Variance: Participant	0.22	0.20	0.20
Variance: Partner (within participant)	0.17	0.17	0.17
Variance: Residual	0.69	0.64	0.64
ICC (participant / partner)	0.20 / 0.16	-	-
**Model Fit**			
**Variance explained**			
Marginal R²	-	0.08	0.08
Conditional R²	-	0.42	0.42
**Fit Statistics**			
AIC	6499.12	6325.02	6325.97
–2LL	6491.12	6299.02	6297.97
**Comparison**			
$\:{\Delta\:}-2LL\:{\chi\:}_{diff}^{2}$Δ − 2LL (χ²)	-	192.10*** (df = 9)	1.05 (df = 1)

Note. Unstandardized estimates (*b*) are reported with standard errors in parentheses. All three models include random intercepts for participants and for partners nested within participants. The focal predictor is the leave-one-out partner–motive residual (see Measures); models are estimated on the 2,430 contact reports for which it could be computed. Reference categories: Gender = Female; Interaction Type = Physical; Regulatory Strategy = Problem Solving. ICC = proportion of the total variance located at the respective level in the null model (participant / partner). AIC = Akaike Information Criterion (lower values indicate better fit); Δ − 2LL tests the significance of model improvement over the preceding model. Parameter estimates and *R*² values refer to the REML fits; AIC, −2LL and all model comparisons refer to the corresponding ML fits. Models with a participant random intercept only and the original Specialization Score are reported in Additional file 1, Table S9.2; the corresponding estimate for the leave-one-out residual is given in Table S9.1. * *p* < .05. ** *p* < .01. *** *p* < .001

Hypothesis 2a predicted that stronger motive-specific pairing would be associated with greater perceived ER effectiveness at the level of single contacts. In the null model, 15% of the variance in perceived ER effectiveness was attributable to stable differences between participants and a further 12% to differences between the specific partners contacted (total ICC = 0.27), which underscores the need to model the partner level explicitly. Including covariates in the baseline model significantly improved model fit (Δ − 2LL = 148.07, df = 9, *p* < .001). Notably, interaction type was a strong predictor: contacts occurring via virtual channels were rated lower than physical contacts (b = − 0.53, SE = 0.06, *p* < .001). Age also emerged as a significant predictor, although the effect size was small (b = 0.02, SE = 0.01, *p* = .004). Among the regulatory motives, humor was associated with higher perceived ER effectiveness than the reference category (problem solving), whereas the other motives did not differ significantly. Contrary to H2a, adding the leave-one-out partner–motive residual did not improve model fit (Δ − 2LL = 0.76, df = 1, *p* = .384), and the fixed effect was not statistically significant (b = − 0.03, SE = 0.03, 95% CI [− 0.08, 0.03], *p* = .383), which corresponds to − 0.02 scale points per standard deviation of the predictor (95% CI [− 0.07, 0.03]). The explanatory power of the fixed effects remained stable (marginal R² = 0.07, conditional R² = 0.31). Consequently, Hypothesis 2a was not supported.

Hypothesis 2b predicted that stronger motive-specific pairing would be associated with a better evaluation of the contact. In the null model, 20% of the variance in interaction evaluation was attributable to participants and 16% to participant-specific partners (total ICC = 0.36). The baseline model significantly improved model fit (Δ − 2LL = 192.10, df = 9, *p* < .001), with covariates showing the same pattern as in H2a. Contrary to expectations, adding the leave-one-out partner–motive residual did not improve model fit (Δ − 2LL = 1.05, df = 1, *p* = .305), and the fixed effect was not statistically significant (b = − 0.02, SE = 0.02, 95% CI [− 0.07, 0.02], *p* = .305), which corresponds to − 0.02 scale points per standard deviation of the predictor (95% CI [− 0.06, 0.02]). The explanatory power of the fixed effects remained stable (marginal R² = 0.08, conditional R² = 0.42) after including the predictor.

The null result proved robust across every alternative specification we examined. Adding EMA network size as a covariate left the focal predictor non-significant for both perceived ER effectiveness (b = − 0.03, *p* = .384) and interaction evaluation (b = − 0.02, *p* = .306), so the null results are not attributable to network size. Reverting to the simpler random-effects structure with a participant intercept only did not change the conclusion either (H2a: b = − 0.01, *p* = .707; H2b: b = − 0.02, *p* = .442), nor did allowing the effect of the predictor to vary randomly across participants (H2a: b = − 0.03, *p* = .336; H2b: b = − 0.03, *p* = .300), the ordinal sensitivity model for perceived ER effectiveness (b = − 0.02, *p* = .714), the exclusion of touch (H2a: b = − 0.01, *p* = .705; H2b: b = − 0.01, *p* = .780), or restricting the models to the three cognitively focused motives (H2a: b = − 0.04, *p* = .378; H2b: b = − 0.04, *p* = .380). The three uncorrected or partly corrected versions of the predictor led to the same conclusion: the original Specialization Score (H2a: b = 0.02, 95% CI [− 0.20, 0.23], *p* = .881; H2b: b = 0.03, 95% CI [− 0.15, 0.20], *p* = .773), its leave-one-out version (H2a: b = 0.03, 95% CI [− 0.18, 0.23], *p* = .814; H2b: b = 0.00, 95% CI [− 0.17, 0.17], *p* > .99) and its lagged version (H2a: b = − 0.003, 95% CI [− 0.17, 0.17], *p* = .973; H2b: b = − 0.04, 95% CI [− 0.18, 0.10], *p* = .575). In the three-level modality specification, the disadvantage of virtual contact was carried primarily by text-based contacts (b = − 0.65, *p* < .001) and was smaller for synchronous virtual contact (b = − 0.27, *p* < .001); the focal predictor remained non-significant (H2a: b = − 0.03, *p* = .394; H2b: b = − 0.02, *p* = .319). The sensitivity analysis using the weighted mean Specialization Score across all listed friends likewise remained non-significant (H2b: b = 0.08, *p* = .389). Full model tables are provided in Additional file 1 (Section S9). Consequently, Hypothesis 2 was not supported.

#### Hypothesis 3: Network specialization as a predictor of well-being and loneliness

Hypothesis 3a predicted that a specialized network predicts higher psychological well-being (for full results, see Table S3, Additional file 1). The baseline model explained a significant amount of variance (*R*² = 0.186, *F*(5, 100) = 4.58, *p* < .001), with number of close friends as the only significant covariate (*B* = 0.83, *SE* = 0.22, *p* < .001). Contrary to H3a, adding the NSI did not improve model fit (ΔR² = 0.001, *F*(1, 99) = 0.13, *p* = .719) and the NSI was not a significant predictor (B = − 1.76, SE = 4.88, 95% CI [− 11.45, 7.93], β = − 0.03, *p* = .719). The full model explained 18.8% of the variance (adjusted R² = 0.138). Assumption checks indicated adequate model fit: residuals showed no substantial violations of linearity and homoscedasticity, multicollinearity was low (VIF < 5), and normality was confirmed via Q-Q plot. Eight influential cases were identified using Cook’s distance; excluding them and refitting both steps did not meaningfully alter the results (Δ*R*² = 0.002, *F*(1, 92) = 0.25, *p* = .619). Consequently, Hypothesis 3a was not supported.

Hypothesis 3b predicted that a specialized network predicts lower loneliness. As shown in Table 5, the baseline model explained a significant amount of variance (*R*² = 0.132, *F*(5, 100) = 3.04, *p* = .013), with fewer close friends significantly associated with higher loneliness (*B* = −1.14, *SE* = 0.38, *p* = .003). Adding the NSI did not significantly increase explained variance (ΔR² = 0.003, *F*(1, 99) = 0.35, *p* = .556), and the NSI was not a significant predictor (B = 4.92, SE = 8.32, 95% CI [− 11.59, 21.43], β = 0.06, *p* = .556). Assumption checks revealed low multicollinearity (VIF < 5) but mild deviations from normality and homoscedasticity. A sensitivity analysis excluding four influential cases (Cook’s distance > 4/*N*; *N* = 102) confirmed that the NSI remained non-significant (*B* = 3.64, *SE* = 7.24, Δ*R*² = 0.002, *F*(1, 95) = 0.25, *p* = .616), suggesting that the absence of a statistically detectable association is robust (see Table S4, Additional file 1).

**Table 5:** Results of hierarchical linear regression predicting social loneliness (*N* = 106)

Predictor	Baseline Model	Full Model
Intercept	28.18 (4.24) ***	25.29 (6.48) ***
**Demographics**		
Age	0.24 (0.15)	0.23 (0.15)
Gender: Male	2.64 (2.69)	2.30 (2.76)
Gender: Other	5.95 (8.06)	6.57 (8.15)
**Network and Context**		
Number of close friends	−1.14 (0.38) **	1.11 (0.39) **
Current stressors (yes vs. no)	−3.07 (3.57)	−2.80 (3.61)
**Main Predictor**		
Network Specialization Index (NSI)		4.92 (8.32)
**Model Fit**		
*R* ^2^	0.132	0.135
*∆R* ^2^	0.132	0.003
F change (df)	3.04 (5, 100)	0.35 (1, 99)
*p*	0.013	0.556

Note. Unstandardized estimates (*B*) are reported with standard errors in parentheses. Reference categories: Gender = Female; Stressors = No. Δ*R*² tests the significance of the increase in variance explained; the degrees of freedom of the corresponding *F* tests are given in parentheses. * *p* < .05. ** *p* < .01. *** *p* < .001

**Fig. 1:**
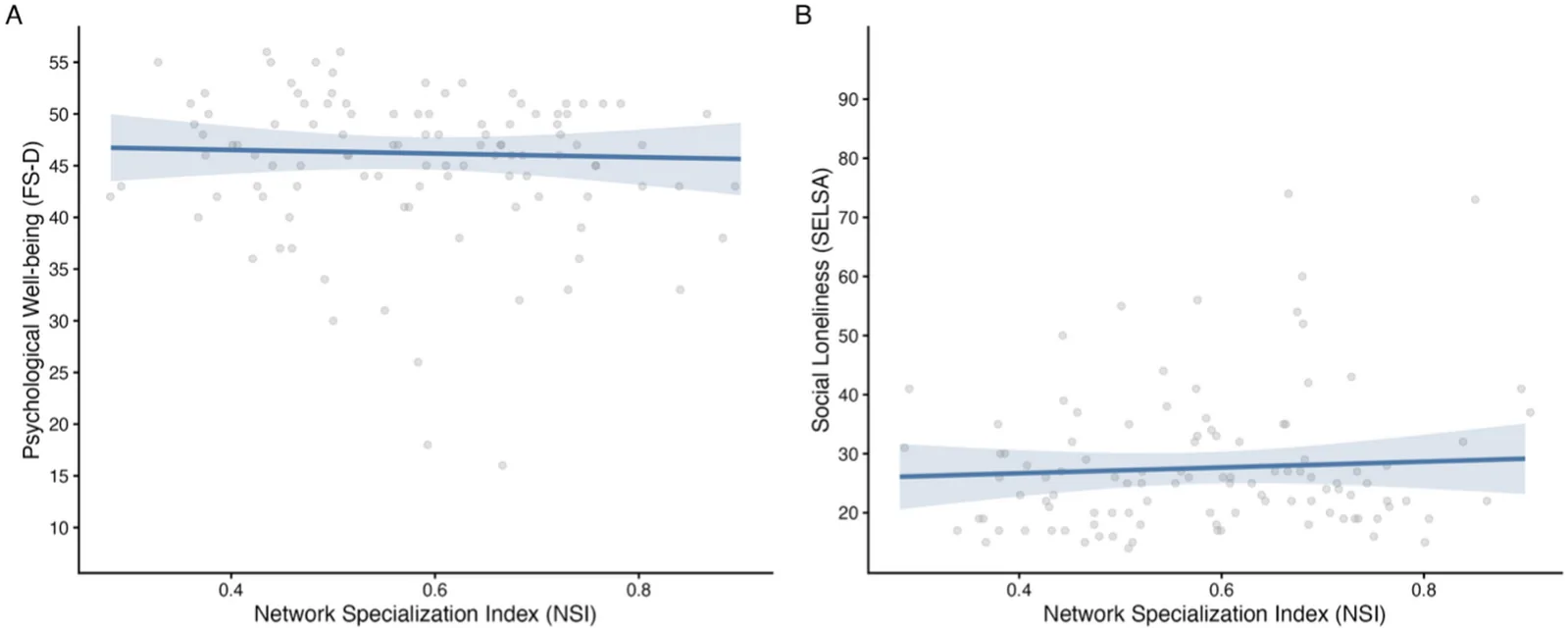
Predicted associations between the Network Specialization Index and (**A**) Psychological Well-being and (**B**) Social Loneliness, with 95% confidence intervals. The lines are model-based predictions from the covariate-adjusted regressions reported in Table 5 and in Additional file 1, Table S3, with the covariates held at their sample means (age = 24.72 years; number of close friends = 5.67) and at their reference categories (gender = female; acute stressors = no). Grey dots represent the raw data

Four further analyses probed these null results. First, parallel models replacing the NSI with the diversity-based indices yielded the same conclusion (FDR: well-being B = − 1.90, 95% CI [− 7.74, 3.95], *p* = .521, ΔR² = 0.003; social loneliness B = 4.83, 95% CI [− 5.12, 14.77], *p* = .338, ΔR² = 0.008; effective number of partners: well-being B = 0.49, 95% CI [− 0.02, 1.00], *p* = .062, ΔR² = 0.028; social loneliness B = − 0.54, 95% CI [− 1.42, 0.34], *p* = .228, ΔR² = 0.013; Additional file 1, Table S8.2). Second, models additionally adjusting for EMA network size and the number of observations per strategy, both of which can mechanically influence specialization scores, left the NSI non-significant (well-being: *B* = 3.96, *p* = .470; loneliness: *B* = − 2.24, *p* = .813; maximum VIF = 1.7). Third, the exploratory network-level partner–motive association index, which quantifies the strength of the association between partner identity and motive rather than the concentration of contact, was likewise unrelated to both outcomes (psychological well-being: B = 0.28, 95% CI [− 0.68, 1.23], *p* = .565, ΔR² = 0.003; social loneliness: B = 0.49, 95% CI [− 1.15, 2.12], *p* = .558, ΔR² = 0.003). Fourth, the primary H3 estimates were small and their confidence intervals included zero as well as associations in both directions (psychological well-being: B = − 1.76, 95% CI [− 11.45, 7.93], β = −0.03; social loneliness: B = 4.92, 95% CI [− 11.59, 21.43], β = 0.06). The sensitivity analysis reported above (minimum detectable increment ΔR² ≈ 7.3%) indicates that the between-person analyses were powered to detect medium-sized increments only. A non-significant result does not by itself exclude smaller associations, and the intervals show that associations of modest size remain compatible with the data. The associations between NSI and both outcomes are visualized in Fig. 1.

## Discussion

Prior research has shown that social relationships, especially friendships, can buffer against distress and are associated with better psychological well-being [30, 34, 49]. Yet, the specific mechanisms of interpersonal emotion regulation remain unclear, including in relation to whom we seek out in our social network and whether the diversity of options influences the outcomes of regulation. By shifting the focus from general social support to functional specificity, the present study examined whether partner–motive pairing, and its network-level aggregate, are associated with better interaction outcomes, higher psychological well-being, and lower social loneliness over a two-week period. To achieve this, EMA was employed to record participants’ friend contacts over two weeks, including the regulatory motive they reported for each contact, the friend they contacted, and how they perceived the contact.

### Discussion of results

The evidence regarding H1 is more nuanced than the preregistered analysis suggested. Against the 1/k benchmark, participants appeared to contact motive-specific “go-to” friends far more often than chance across all six motives. Under participant-level permutation nulls that preserve individual contact patterns, however, this apparent effect largely disappeared: no motive exceeded the permutation null after Holm correction, and only a small minority of participants (4.6–14.3%) exceeded their individual null. An omnibus test at the network level, which pools the evidence across motives instead of testing each motive separately, did detect a small association above chance on average (d = 0.36), significant for 18 of the 104 participants for whom the index could be computed. Motive-specific pairing is therefore present in these data, but it is weak, it is carried by a minority of participants, and it is largely invisible in the motive-by-motive comparisons that the preregistered hypothesis specified. The residualized specialization metric added a complementary nuance: problem solving (and, to a lesser degree, distraction) was channeled toward specific friends beyond their overall involvement, whereas humor was not. Two conclusions follow. First, much of what looks like functional specialization in raw selection data reflects the general concentration of social contact on few partners, a pattern consistent with, but not specific to, strategic partner selection. Second, where motive-specific channeling does emerge, it does so selectively, for problem solving and, more weakly, for distraction under the residualized metric, rather than across the board. Touch showed the numerically largest deviation under the original metric, but it did not survive correction for multiple testing (Holm-corrected *p* = .061), it was not supported by the residualized analysis (Holm-corrected *p* = .496; Additional file 1, Table S7.1), and only ten participants contributed at least three touch episodes. We therefore do not treat touch as evidence of motive-specific channeling, and any normative interpretation of touch would be speculation that the present analyses do not support. Our data therefore demonstrate recurrent partner–motive pairing; they do not yet establish motive-specific partner competence or causal regulatory benefits. Contrary to expectations, H2 was not supported. This result remained robust when controlling for EMA network size, which makes it unlikely that the focal predictor merely reflected a structural artifact of small networks. It also was robust against every additional specification we examined: the simpler participant-only random-effects structure, random slopes, the uncorrected and the partly corrected versions of the predictor, ordinal models, modality refinements, and motive subsets. Instead, interaction type emerged as the strongest predictor, with physical interactions rated higher than virtual ones; the refined modality analysis further showed that this disadvantage was carried primarily by text-based contact, whereas synchronous virtual contact (phone/video) fell in between. Humor was the strategy most consistently associated with better outcomes, likely due to its immediate mood-enhancing properties [50]. The advantage of in-person contact should not be over-interpreted as a modality effect per se: it may equally reflect closeness, interaction duration, shared activities, immediacy, or selection into in-person contact.

### Selection versus efficacy: why partner–motive pairing may not translate into regulatory benefits

The clearest theoretical implication of these results is a distinction between selection and efficacy. Participants showed recurrent partner–motive pairing, and a small part of it exceeded what their overall contact pattern implies, yet the strength of the pairing showed no detectable association with how the contact was experienced. Four mechanisms could produce selection without efficacy gains. First, habit and relational scripts: partner choice in daily life is largely routine and is maintained by repetition rather than by outcome feedback, so a pairing can persist without being the one that works best [25]. Second, availability and proximity: Social Baseline Theory holds that the presence of a trusted other is itself regulatory [3, 20], which predicts that whoever is at hand will do and that benefits accrue from contact rather than from matching. Third, shared situational context: partners are often present precisely in the situations that generate a given motive, such as a flatmate when distraction is sought, so that pairing may be a by-product of co-presence. Fourth, relational rather than regulatory reward: contacts may be repeated because they are enjoyable and affirm the relationship, which is consistent with the uniformly favorable interaction evaluations observed here. Perceived Partner Responsiveness predicts benefits when the response matches the need [26, 27]. The present null results do not contradict that prediction, because we recorded which partner was contacted for which motive rather than whether the response actually matched the need. Finally, whether matching matters may depend on the type and intensity of the emotion being regulated, which the present design, assessing motives but not discrete emotions, could not resolve. Separating selection from efficacy therefore requires designs that record what partners do and not only who is contacted: assessments of the partner’s response, momentary affect before and after contact, or experimental assignment of regulation partners.

Hypothesis 3 was not supported. The NSI did not explain significant unique variance in either psychological well-being or social loneliness beyond the baseline covariates. A plausible objection is that this null result stems from the construction of the index itself: the NSI quantifies the average strength of preference for a go-to friend per strategy but cannot distinguish a differentiated network (different specialists per strategy) from reliance on a single friend across all strategies. We tested this possibility directly. The NSI and the Functional Differentiation Ratio were only weakly correlated, confirming that concentration and differentiation are distinct constructs. Yet neither the FDR nor the effective number of partners predicted well-being or loneliness. The null result at the network level is therefore unlikely to be an artifact of the NSI’s construction, although the modest between-person power means that small associations cannot be ruled out. It should nevertheless be stated plainly that none of these three indices tests functional specialization as originally theorized. The NSI is a concentration index, the FDR is a coarse count of how many distinct partners hold a maximum, and the effective number of partners describes how broadly contact is spread. None of them quantifies the strength of the association between partner identity and regulatory motive at the network level. We therefore added an index that does: the participant-level partner × motive association, standardized against each participant’s own permutation null and thereby adjusted for network size and for how often each motive was reported. This index behaved as intended, in that it exceeded chance on average, but it was no more closely related to psychological well-being or to social loneliness than the concentration and diversity indices were. The network-level null result is therefore not an artifact of how specialization was indexed: neither the concentration of everyday friend contact, nor its breadth, nor the strength of the partner–motive association itself was associated with the two outcomes in this sample. Because this index was not preregistered, the corresponding analyses are exploratory. An alternative account is that individuals with more specialized networks face greater regulatory demands, which could mask benefits of specialization. As a coarse probe, the NSI was essentially unrelated to the presence of acute stressors during the study period. A conclusive test would, however, require momentary assessments of regulatory demand, which we highlight as a direction for future research. These results should finally be read against what is known about the structure of everyday friendship networks. Participants named on average 8.90 partners across two weeks but concentrated their contacts on a small subset of them. Network research indicates that such concentration is typical: friendship networks differ in whether they form one cohesive cluster or several clusters attached to different life domains [51], and friendship network types differ systematically in size and composition across adulthood [52]. If most regulatory contacts are distributed over a few frequently contacted partners, the structural scope for motive-level differentiation is limited, which offers a parsimonious account of both the limited evidence for motive-specific channeling and the absence of network-level associations. We note that popular claims about an optimal number of friends are not currently supported by evidence of sufficient rigor to serve as a benchmark, and we therefore do not anchor this interpretation to a specific network size.

### Limitations

Several limitations of the study should be acknowledged. First, and most importantly, the study measured reported partner–motive pairing rather than interpersonal emotion regulation as theorized. Participants were not screened for a regulatory need before the motive item, the response the friend actually gave was not assessed, and several contacts within one interval were aggregated into a single report. This applies both to repeated contacts with the same friend and to reports naming more than one friend (16.5% of reports), which the models assign to the first-named friend. The observations therefore establish associations between reported contact partners and hoped-for benefits. They do not establish that a partner was selected because of a regulatory need, that the partner provided the corresponding strategy, or that regulation took place at all. All interpretations offered here are framed accordingly, and terms such as functional specialization refer to a theoretical construct that the present design can only approximate. Second, the operationalization of the Specialization Score requires critical examination. As a novel metric constructed specifically for this study, it lacks established validation and is subject to inherent structural constraints. A closer analysis suggests that the score may capture consistency of involvement rather than true functional specialization. For example, if a participant tends to contact a best friend frequently – independently of the specific regulatory need – this would inflate that friend’s Specialization Score. In such cases, the score may reflect general closeness or availability rather than motive-specific competence. The permutation and residualization analyses address this concern empirically and confirm that it was warranted: much of the raw “go-to” pattern is attributable to overall involvement. The corrected predictor used in the primary H2 analyses removes this confound, but it can only be computed when a motive or a partner appears more than once, so that 13.1% of contact reports, and two participants, do not contribute to those models. We retain this limitation because even the residualized metric cannot establish that friends were selected for motive-specific competence.

Third, the assessment of the IER repertoire was constrained by the study design. Instead of assessing the full individual IER repertoire through free-text responses, the study employed a forced-choice format limited to six preselected strategies. While this standardization facilitated quantitative comparison, it inevitably simplified the complexity of an individual’s regulation repertoire. This approach likely excluded strategies specific to individual friendships, potentially failing to capture the full variance of the participants' actual regulatory behavior. Relatedly, the single-choice motive item did not include an opt-out option, so contacts without any affect-improving motive could not be identified; reports in the humor and distraction categories, in particular, may partly reflect everyday sociability rather than intentional interpersonal emotion regulation. Consistent with this concern, sensitivity analyses excluding humor and distraction yielded no evidence of motive-specific pairing beyond the permutation null for the remaining strategies (Additional file 1, Table S6.2). Moreover, because momentary affect was not assessed, we could not restrict analyses to emotionally salient interactions or examine whether specialization effects depend on the valence and intensity of the regulated emotion.

Fourth, “friend” was participant-defined: although the EMA prompt restricted reports to friends, the relationship type of EMA partners was not independently verified, and the composition of the EMA network by relationship category therefore cannot be reported. Some named “friends” may occupy hybrid roles (e.g., romantic partners or roommates who are also considered friends). Because relationship type was not recorded, we cannot state what proportion of nominated partners were conventional friends rather than romantic partners, relatives, flatmates, or colleagues who are also regarded as friends. Given that friendship is central to the framing and the title of this study, this is a substantive rather than a technical limitation: the findings describe contacts with self-defined friends, and their generalization to friendship as a distinct relationship category requires studies that record relationship type for every nominated partner.

Fifth, because people choose whom they turn to, naturally occurring networks contain little variance in poorly matched regulation attempts; this range restriction limits our ability to detect benefits of matching. Experimental designs assigning participants to well- versus poorly matched regulators would provide a stronger test and are a critical next step

Finally, although EMA enhances ecological validity by capturing real-world data, it also faces inherent methodological challenges. Unlike laboratory settings, EMA lacks control over extraneous variables that may influence results. In the present study, the design prioritized minimizing participant burden and drop-out, which caused two key restrictions: First, instructions were short to ensure brevity, which may have compromised clarity, resulting in data irregularities such as the inconsistent labeling of network members. Second, excluding detailed covariates, such as affect changes and relationship depth, was necessary to maintain short survey completion. Although practical, single-item measures of ER effectiveness are additionally prone to measurement errors [53]. Despite these limitations, EMA remains one of the best available methods for studying interactions in real life.

Beyond these methodological constraints, the generalizability of findings is limited by the reliance on a WEIRD (Western, Educated, Industrialized, Rich, Democratic) sample, consisting primarily of young university students [54, 55]. This demographic restricts external validity, because especially in young university students the role of friendships and social networks differs from older adults or non-student populations [56].

## Conclusions

Overall, our results point to a discrepancy between behavioral preferences and psychological outcomes and to a more parsimonious reading of the behavioral preferences themselves. Behaviorally, participants repeatedly paired specific partners with specific regulatory motives, but this pairing largely mirrored their overall contact patterns. Motive-specific channeling beyond general involvement emerged only for a subset of motives and was small when aggregated across the network. This qualifies, rather than confirms, an extension of the Emotionship framework [11] to the level of regulatory motives. Yet, at the level of individual contacts, neither perceived ER effectiveness nor interaction evaluation showed a statistically detectable association with partner-specific pairing in any model specification. This indicates that no association was detected, not that none exists. Whether the benefits of social contact instead derive from availability and presence, as Social Baseline Theory suggests [3], remains to be tested directly. Moreover, the analyses of psychological well-being and social loneliness underscore the distinction between network indices derived from self-reported contact reports and the subjective experience of support [57]. This distinction may help explain why the NSI and the diversity-based indices examined alongside it showed no statistically detectable associations with psychological outcomes, and it suggests that the association between partner identity and regulatory motive, which we indexed directly in an exploratory analysis, is not in itself what links a friendship network to psychological adjustment.

## Supplementary Information

Below is the link to the electronic supplementary material.

Supplementary Material 1: Additional file 1: Supplementary tables and analyses Table S1-S11

## Data Availability

The hypotheses and analysis plan for this study were preregistered at OSF prior to data collection. The preregistration, scripts and datasets generated and analyzed during the current study are publicly available at https://doi.org/10.17605/OSF.IO/MBJRP.

## Abbreviations

CI: confidence interval
EMA: ecological momentary assessment
ER: emotion regulation
FDR: Functional Differentiation Ratio
FS-D: Flourishing Scale, German version
ICC: intraclass correlation coefficient
IER: interpersonal emotion regulation
ML: maximum likelihood
NSI: Network Specialization Index
OSF: Open Science Framework
REML: restricted maximum likelihood
SELSA: Social and Emotional Loneliness Scale for Adults
VIF: variance inflation factor
