# Supplementary material for: FRIENDS – friendship-based regulation of individual emotions in networks in daily situations: An EMA study on partner–motive pairing and social outcomes in adult friendships
Source: BMC Psychol. 2026 Oct 14;14:1477. doi: 10.1186/s40359-026-05767-w (PMC13670613; doi:10.1186/s40359-026-05767-w)
Supplement: Supplementary file 1 [file 40359_2026_5767_MOESM1_ESM.docx]

**Additional file 1**

**Supplementary tables and analyses for**

**FRIENDS – Friendship-based Regulation of Individual Emotions in Networks in Daily Situations: An EMA Study on Partner–Motive Pairing and Social Outcomes in Adult Friendships**

**Table S1a.** Results of the Preregistered One-Tailed Paired-Samples t-Tests (Maximum Specialization Score vs. 1/k).

| **Strategy** | **n** | **MSS M (SD)** | **ECP M (SD)** | **t (df)** | **p (Holm)** | **Cohen's d** |
| --- | --- | --- | --- | --- | --- | --- |
| Problem Solving | 105 | 0.56 (0.23) | 0.14 (0.07) | 19.61 (104) | < .001 | 1.91 |
| Encouragement | 72 | 0.70 (0.27) | 0.14 (0.07) | 17.68 (71) | < .001 | 2.08 |
| Reappraisal | 80 | 0.61 (0.25) | 0.14 (0.08) | 17.78 (79) | < .001 | 1.99 |
| Humor | 103 | 0.50 (0.24) | 0.14 (0.07) | 16.92 (102) | < .001 | 1.67 |
| Distraction | 103 | 0.49 (0.22) | 0.14 (0.07) | 17.71 (102) | < .001 | 1.75 |
| Touch | 47 | 0.83 (0.26) | 0.13 (0.09) | 18.45 (46) | < .001 | 2.69 |

*Note.* These are the preregistered analyses, retained for transparency. As detailed in the main text (Statistical Analyses), the 1/k benchmark is inflated by an order-statistics artifact; the primary test of Hypothesis 1 is the participant-level permutation analysis (Table 3, main text). MSS = maximum Specialization Score; ECP = expected contact probability (1/k). Each strategy was tested only with those participants who used it at least once, which is why the sample sizes exceed those in Table 3 of the main text (there, participants contributed to a strategy only with at least three occasions of that strategy). p-values are Bonferroni–Holm corrected across the six strategies.

**Table S1b.** Results of the Wilcoxon Signed-Rank Tests (Non-Parametric Complement to Table S1a).

| **Strategy** | **n** | **V** | **p (Holm)** | **r** |
| --- | --- | --- | --- | --- |
| Problem Solving | 105 | 5,565 | < .001 | .87 |
| Encouragement | 72 | 2,628 | < .001 | .87 |
| Reappraisal | 80 | 3,160 | < .001 | .86 |
| Humor | 103 | 5,356 | < .001 | .87 |
| Distraction | 103 | 5,356 | < .001 | .87 |
| Touch | 47 | 1,081 | < .001 | .86 |

*Note.* One-tailed Wilcoxon signed-rank tests comparing maximum Specialization Scores against the expected contact probability. In every strategy all difference scores were positive, so V attains its maximum value n(n + 1)/2; the identical values for humor and distraction follow from their identical sample size (n = 103) and are not a transcription error. Effect sizes were computed as r = |Z|/√n from the normal approximation of the test statistic. p-values are Bonferroni–Holm corrected across the six strategies.

**Table S2.** Results of the Hierarchical Linear Mixed Models Predicting Perceived ER Effectiveness (H2a; 2,430 Contact Reports from 104 Participants and 508 Participant-Specific Partners).

| **Predictor** | **Null Model** | **Baseline Model** | **Full Model** |
| --- | --- | --- | --- |
| Intercept | 5.60 (.06) *** | 5.70 (.08) *** | 5.70 (.08) *** |
| **Level-2 covariates** |  |  |  |
| Age |  | 0.02 (.01) ** | 0.02 (.01) ** |
| Gender: Male |  | −0.34 (.14) * | −0.34 (.14) * |
| Gender: Other |  | −0.40 (.40) | −0.40 (.40) |
| **Level-1 covariates** |  |  |  |
| Interaction type: Virtual |  | −0.53 (.06) *** | −0.53 (.06) *** |
| Strategy: Encouragement |  | 0.14 (.10) | 0.15 (.10) |
| Strategy: Reappraisal |  | −0.09 (.09) | −0.09 (.09) |
| Strategy: Humor |  | 0.32 (.07) *** | 0.32 (.07) *** |
| Strategy: Distraction |  | 0.10 (.07) | 0.10 (.07) |
| Strategy: Touch |  | −0.13 (.15) | −0.12 (.15) |
| **Main predictor** |  |  |  |
| Partner–motive residual (leave-one-out) |  |  | −0.03 (.03) |
| **Random effects** |  |  |  |
| Variance: Participant | 0.22 | 0.19 | 0.19 |
| Variance: Partner (within participant) | 0.18 | 0.18 | 0.18 |
| Variance: Residual | 1.09 | 1.03 | 1.03 |
| ICC (participant / partner) | .15 / .12 | – | – |
| **Variance explained** |  |  |  |
| Marginal R² | – | .07 | .07 |
| Conditional R² | – | .31 | .31 |
| **Fit statistics** |  |  |  |
| AIC (ML) | 7500.35 | 7370.28 | 7371.52 |
| −2LL (ML) | 7492.35 | 7344.28 | 7343.52 |
| Δ−2LL (χ²) | – | 148.07*** (df = 9) | 0.76 (df = 1) |

*Note.* Unstandardized estimates (b) with standard errors in parentheses. This is the primary specification: the leave-one-out partner–motive residual as the focal predictor (see Measures in the main text) and random intercepts for participants and for partners nested within participants, which are nested rather than crossed because each partner belongs to exactly one participant. Models are estimated on the 2,430 contact reports for which the focal predictor could be computed. Reference categories: Gender = female; interaction type = physical; regulatory motive = problem solving. ICC = proportion of the total variance located at the respective level in the null model. Parameter estimates and R² values refer to the REML fits; AIC, −2LL and all model comparisons refer to the corresponding ML fits. The effect of the focal predictor is b = −0.026, SE = 0.030, 95% CI [−0.084, 0.032], p = .383. Estimates for the original Specialization Score are summarized in Table S9.1; models with a participant random intercept only (original Specialization Score) are reported in Table S9.2. * p < .05. ** p < .01. *** p < .001.

**Table S3.** Results of the Hierarchical Linear Regression Predicting Psychological Well-Being (H3a; N = 106).

| **Predictor** | **Baseline Model** | **Full Model** |
| --- | --- | --- |
| Intercept | 40.34 (2.48) *** | 41.38 (3.80) *** |
| **Demographics** |  |  |
| Age | 0.05 (0.09) | 0.05 (0.09) |
| Gender: Male | −3.07 (1.58) | −2.95 (1.62) |
| Gender: Other | −8.89 (4.72) | −9.12 (4.78) |
| **Network and context** |  |  |
| Number of close friends | 0.83 (0.22) *** | 0.82 (0.23) *** |
| Current stressors (yes vs. no) | −0.93 (2.09) | −1.02 (2.12) |
| **Main predictor** |  |  |
| Network Specialization Index (NSI) | – | −1.76 (4.88) |
| **Model fit** |  |  |
| R² | .186 | .188 |
| ΔR² | .186 | .001 |
| F change (df) | 4.58 (5, 100) | 0.13 (1, 99) |
| p | < .001 | .719 |

*Note.* Unstandardized estimates (B) with standard errors in parentheses. Reference categories: Gender = female; stressors = no. ΔR² tests the significance of the increase in variance explained; the degrees of freedom of the corresponding F tests are given in parentheses. The baseline model contains five predictor columns (close friends, stressors, age, and two gender dummies), hence F(5, 100). Excluding the eight high-influence cases identified by Cook’s distance and refitting both steps left the NSI non-significant (B = −2.13, SE = 4.27, ΔR² = .002, F(1, 92) = 0.25, p = .619; N = 98). * p < .05. ** p < .01. *** p < .001.

**Table S4.** Sensitivity Analysis for Social Loneliness (H3b): Hierarchical Linear Regression Excluding High-Influence Cases (N = 102).

| **Predictor** | **Baseline Model** | **Full Model** |
| --- | --- | --- |
| Intercept | 32.95 (4.81) *** | 31.07 (6.11) *** |
| **Demographics** |  |  |
| Age | −0.10 (0.19) | −0.11 (0.19) |
| Gender: Male | 0.92 (2.40) | 0.74 (2.43) |
| Gender: Other | 6.16 (6.85) | 6.65 (6.95) |
| **Network and context** |  |  |
| Number of close friends | −0.65 (0.33) | −0.63 (0.34) |
| Current stressors (yes vs. no) | −4.20 (3.17) | −4.00 (3.21) |
| **Main predictor** |  |  |
| Network Specialization Index (NSI) | – | 3.64 (7.24) |
| **Model fit** |  |  |
| R² | .078 | .081 |
| ΔR² | .078 | .002 |
| F change (df) | 1.64 (5, 96) | 0.25 (1, 95) |
| p | .158 | .616 |

*Note.* Outcome: social loneliness (SELSA). Four cases with Cook’s distance > 4/N were excluded and both model steps were refitted in the reduced sample. Unstandardized estimates (B) with standard errors in parentheses. Reference categories: Gender = female; stressors = no. The corresponding full-sample model is Table 5 in the main text. * p < .05. ** p < .01. *** p < .001.

**Table S5.** Descriptions of the Six Affect-Improving Interpersonal Emotion Regulation Strategies Investigated in the Present Study.

| **Strategy** | **Description** |
| --- | --- |
| Problem Solving | A problem-focused strategy involving direct action to address the cause of distress (e.g., brainstorming solutions, planning a course of action). Regulates affect indirectly by modifying or eliminating the external stressor (Marroquín et al., 2017). |
| Encouragement | Regulates emotion by shifting focus from the problem to the individual, drawing attention to the target’s positive characteristics or past successes to strengthen their self-concept (Wong et al., 2025). Also referred to as social validation. |
| Reappraisal | Reduces the negative impact of a situation by changing its interpretation. Occurring early in the emotion-generation process, interpersonal reappraisal can prevent negative emotions from escalating (Gross & John, 2003) and has been shown to enhance mood and emotional well-being (Horn & Maercker, 2016). |
| Humor | Introduces positive affect and creates cognitive distance from a stressor through lighthearted reframing. Shared laughter enhances positive affect and relationship quality (Kashdan et al., 2014) and can outperform rational reappraisal in down-regulating negative emotions (Kugler & Kuhbandner, 2015). |
| Distraction | Diverts attention from the stressor through shared activities or conversation topic shifts. Differs from suppression by introducing positive or neutral alternatives (Gross, 2002). Adaptive when used for temporary distance, but potentially maladaptive if used for habitual avoidance (Wolgast & Lundh, 2017). |
| Touch | Physical contact as a nonverbal regulation strategy. In romantic relationships, responsive touch reliably enhances the partner’s positive affect (Debrot et al., 2013). Its regulatory function in friendships remains less empirically established, though socially normative touch (e.g., a supportive hug) is theoretically well-motivated (Suvilehto et al., 2015). |

*Note.* All strategies belong to the affect-improving class of Niven et al.’s (2009) taxonomy. The response options by which the six motives were presented to participants are reported in Table 1 of the main text.

**S6. Permutation Analyses for Hypothesis 1: Method Details and Sensitivity Variants**

For each participant, strategy labels were permuted across that participant’s reported contacts (5,000 iterations). Because friend sets remain attached to their contacts, this procedure preserves (a) the participant’s number of occasions per strategy and (b) each friend’s total (weighted) involvement, i.e. both margins of the friend × strategy table. For each strategy, the observed maximum Specialization Score (MSS) was compared with its permutation null distribution, yielding participant-level empirical p-values, (1 + #{null ≥ observed})/(iterations + 1). Group-level tests compared the observed mean MSS against the permutation distribution of the mean; z = (observed mean − mean of null means)/SD of null means. Participants contributed to a strategy’s test only with at least three occasions of that strategy. The full analysis script is available on OSF.

**Table S6.1.** Hypothesis 1 Permutation Tests Excluding Touch.

| **Strategy** | **n** | **M(obs)** | **M(null)** | **z** | **p perm** | **p (Holm)** |
| --- | --- | --- | --- | --- | --- | --- |
| Problem Solving | 70 | 0.486 | 0.460 | 1.59 | .055 | .165 |
| Encouragement | 35 | 0.544 | 0.483 | 2.42 | .008 | .039 |
| Reappraisal | 42 | 0.524 | 0.469 | 2.26 | .014 | .056 |
| Humor | 87 | 0.460 | 0.450 | 0.75 | .235 | .416 |
| Distraction | 92 | 0.439 | 0.430 | 0.83 | .208 | .416 |

*Note.* Permutations were recomputed within the reduced strategy pool. Only encouragement exceeded the null after Holm correction, and it did so marginally; the pattern otherwise matches the full six-motive analysis (Table 3, main text).

**Table S6.2.** Hypothesis 1 Permutation Tests Restricted to the Cognitively Focused Strategies and Touch (Excluding Humor and Distraction).

| **Strategy** | **n** | **M(obs)** | **M(null)** | **z** | **p perm** | **p (Holm)** |
| --- | --- | --- | --- | --- | --- | --- |
| Problem Solving | 70 | 0.486 | 0.491 | −0.34 | .632 | .632 |
| Encouragement | 35 | 0.544 | 0.510 | 1.42 | .080 | .241 |
| Reappraisal | 42 | 0.524 | 0.499 | 1.06 | .145 | .291 |
| Touch | 10 | 0.612 | 0.512 | 1.98 | .025 | .101 |

*Note.* Because permutations redistribute strategy labels within the reduced strategy pool, these analyses are not directly comparable with the full six-motive analysis (Table 3, main text); they serve as a sensitivity check of the interpersonal-emotion-regulation interpretation, i.e. whether motive-specific pairing is still detectable once the categories most likely to reflect everyday sociability are removed. No strategy exceeded the null after Holm correction.

**S7. Residualized Specialization Metric**

For each participant, standardized Pearson residuals were computed from the weighted friend × strategy contingency table: r = (observed − expected)/√expected, with expected values derived from the table margins under independence. Positive residuals indicate that a friend was contacted for a given strategy more often than expected from that friend’s overall contact frequency. The maximum residual per strategy was tested against its permutation null (same procedure as in S6).

**Table S7.1.** Permutation Tests of the Maximum Standardized Residual per Strategy.

| **Strategy** | **n** | **M(obs)** | **M(null)** | **z** | **p perm** | **p (Holm)** |
| --- | --- | --- | --- | --- | --- | --- |
| Problem Solving | 70 | 1.55 | 1.36 | 3.29 | < .001 | .005 |
| Encouragement | 35 | 1.65 | 1.54 | 1.16 | .124 | .496 |
| Reappraisal | 42 | 1.66 | 1.58 | 0.86 | .190 | .496 |
| Humor | 87 | 1.20 | 1.17 | 0.75 | .223 | .496 |
| Distraction | 92 | 1.18 | 1.09 | 2.44 | .008 | .039 |
| Touch | 10 | 1.87 | 1.66 | 1.13 | .136 | .496 |

*Note.* Strategy-specific channeling, i.e. friends being contacted for a strategy beyond their overall involvement, emerged for problem solving and distraction only. p perm for problem solving is .0008. p-values are Bonferroni–Holm corrected across the six strategies.

**S8. Decomposition of the Network Specialization Index: Diversity Indices and H3 Reruns**

The Functional Differentiation Ratio (FDR) was computed as the number of distinct primary partners divided by the number of motives reported; the effective number of partners was computed as exp(H), where H is the Shannon entropy of a participant’s weighted contact distribution across friends.

**Table S8.1.** Correlations Among the Network Indices (N = 106).

|  | **NSI** | **FDR** | **Effective number of partners** | **EMA network size** |
| --- | --- | --- | --- | --- |
| NSI | – | −.30 | −.63 | −.52 |
| FDR | −.30 | – | .49 | .31 |
| Effective number of partners | −.63 | .49 | – | .87 |
| EMA network size | −.52 | .31 | .87 | – |

*Note.* Pearson correlations. The network-level partner–motive association index correlates .18 with the NSI, .41 with the FDR, −.07 with the effective number of partners and −.03 with EMA network size.

**Table S8.2.** Hierarchical Regressions Predicting Well-Being and Loneliness With Alternative Network Indices.

| **Outcome** | **Predictor** | **B** | **SE** | **p** | **ΔR²** |
| --- | --- | --- | --- | --- | --- |
| Well-being | NSI | −1.76 | 4.88 | .719 | .001 |
| Well-being | FDR | −1.90 | 2.94 | .521 | .003 |
| Well-being | Effective number of partners | 0.49 | 0.26 | .062 | .028 |
| Loneliness | NSI | 4.92 | 8.32 | .556 | .003 |
| Loneliness | FDR | 4.83 | 5.01 | .338 | .008 |
| Loneliness | Effective number of partners | −0.54 | 0.45 | .228 | .013 |

*Note.* Each row reports the increment of the focal index over the covariate baseline (number of close friends, current stressors, age, gender); all F tests for the increment have (1, 99) degrees of freedom. The 95% confidence intervals of the estimates are, in the order of the rows, [−11.45, 7.93], [−7.74, 3.95], [−0.02, 1.00], [−11.59, 21.43], [−5.12, 14.77] and [−1.42, 0.34]. The exploratory network-level partner–motive association index yielded B = 0.28, 95% CI [−0.68, 1.23], p = .565, ΔR² = .003 for well-being and B = 0.49, 95% CI [−1.15, 2.12], p = .558, ΔR² = .003 for social loneliness (n = 104). Models additionally adjusting for EMA network size and observations per strategy yielded the same conclusion (NSI: well-being B = 3.96, p = .470; loneliness B = −2.24, p = .813; maximum VIF = 1.7).

**S9. Robustness of the Hypothesis 2 Models**

The primary specification reported in Table 4 (main text) and Table S2 uses the leave-one-out partner–motive residual as the focal predictor and random intercepts for participants and for partners nested within participants. Adding the partner intercept to the participant-only model improved fit for both outcomes (perceived ER effectiveness: χ²(1) = 73.05, p < .001, AIC 7442.57 → 7371.52; interaction evaluation: χ²(1) = 133.18, p < .001, AIC 6457.15 → 6325.97), which is why the partner-level model is designated as primary. A random slope for the focal predictor did not improve fit for either outcome (perceived ER effectiveness: χ²(2) = 2.41, p = .300; interaction evaluation: χ²(2) = 2.05, p = .358), and neither random-slope model was singular or produced a convergence warning, so the random-intercept structure is retained on statistical grounds. Table S9.1 collects the within-person effect of the focal predictor and of the three versions of the original Specialization Score across all specifications we examined; Table S9.2 reports the full participant-only models with the original score.

**Table S9.1. Within-Person Effect of the Focal Predictor on the Hypothesis 2 Outcomes Across Specifications.**

| **Specification** | **H2a: Perceived ER effectiveness b (p)** | **H2b: Interaction evaluation b (p)** |
| --- | --- | --- |
| **Primary model: leave-one-out partner–motive residual (participant + partner random intercepts; Table 4 / Table S2)** | **−0.03 (.383)** | **−0.02 (.305)** |
| + EMA network size as covariate | −0.03 (.384) | −0.02 (.306) |
| + random slope for the focal predictor | −0.03 (.336) | −0.03 (.300) |
| Participant random intercept only | −0.01 (.707) | −0.02 (.442) |
| Excluding touch | −0.01 (.705) | −0.01 (.780) |
| Restricted to the cognitively focused motives | −0.04 (.378) | −0.04 (.380) |
| Three-level modality factor | −0.03 (.394) | −0.02 (.319) |
| Cumulative link mixed model (ordinal outcome) | −0.02 (.714) | – |
| Original Specialization Score | 0.02 (.881) | 0.03 (.773) |
| Leave-one-out Specialization Score | 0.03 (.814) | 0.00 (> .99) |
| Lagged Specialization Score, prior contact reports only | −0.003 (.973) | −0.04 (.575) |
| Weighted mean Specialization Score across all listed friends | – | 0.08 (.389) |

*Note. Unless stated otherwise, all specifications use the primary random-effects structure. The focal predictor is the leave-one-out partner–motive residual; the four bottom rows report the original Specialization Score and its corrected versions for comparison. Usable contact reports: focal predictor n = 2,430; leave-one-out score n = 2,703; lagged score n = 2,286; all other specifications n = 2,430 unless a subgroup is named (excluding touch n = 2,360; cognitively focused motives n = 814). With the original Specialization Score, a random slope improved fit for perceived ER effectiveness (χ²(2) = 6.50, p = .039) but not for interaction evaluation (χ²(2) = 4.43, p = .109); with the focal predictor it improved neither.*

**Table S9.2.** Sensitivity Analysis: Hypothesis 2 Models With a Participant Random Intercept Only.

| **Predictor** | **H2a Baseline** | **H2a Full** | **H2b Baseline** | **H2b Full** |
| --- | --- | --- | --- | --- |
| Intercept | 5.66 (.08) *** | 5.66 (.08) *** | 5.86 (.07) *** | 5.86 (.07) *** |
| **Level-2 covariates** |  |  |  |  |
| Age | 0.02 (.01) ** | 0.02 (.01) ** | 0.02 (.01) ** | 0.02 (.01) ** |
| Gender: Male | −0.30 (.12) * | −0.30 (.12) * | −0.20 (.12) | −0.20 (.12) |
| Gender: Other | −0.29 (.39) | −0.29 (.39) | −0.20 (.38) | −0.20 (.38) |
| **Level-1 covariates** |  |  |  |  |
| Interaction type: Virtual | −0.44 (.05) *** | −0.44 (.05) *** | −0.44 (.04) *** | −0.44 (.04) *** |
| Strategy: Encouragement | 0.05 (.09) | 0.03 (.09) | 0.09 (.07) | 0.07 (.07) |
| Strategy: Reappraisal | −0.05 (.09) | −0.06 (.09) | −0.03 (.07) | −0.05 (.07) |
| Strategy: Humor | 0.31 (.07) *** | 0.31 (.07) *** | 0.21 (.05) *** | 0.21 (.05) *** |
| Strategy: Distraction | 0.11 (.07) | 0.12 (.07) | −0.04 (.05) | −0.03 (.05) |
| Strategy: Touch | −0.11 (.13) | −0.14 (.13) | −0.10 (.10) | −0.14 (.10) |
| **Main predictor** |  |  |  |  |
| Specialization Score |  | 0.10 (.10) |  | 0.13 (.08) |
| **Random effects** |  |  |  |  |
| Variance: Participant | 0.21 | 0.21 | 0.22 | 0.22 |
| Variance: Residual | 1.19 | 1.19 | 0.77 | 0.77 |
| ICC (null model) | .16 | .16 | .23 | .23 |
| **Variance explained** |  |  |  |  |
| Marginal R² | .05 | .05 | .06 | .06 |
| Conditional R² | .20 | .20 | .27 | .27 |
| **Fit statistics** |  |  |  |  |
| AIC (ML) | 8624.52 | 8625.53 | 7453.72 | 7453.03 |
| −2LL (ML) | 8600.52 | 8599.53 | 7429.72 | 7427.03 |
| Δ−2LL (χ²) | 130.06*** (df = 9) | 0.99 (df = 1) | 162.98*** (df = 9) | 2.68 (df = 1) |

*Note.* Unstandardized estimates (b) with standard errors in parentheses; random intercept for participants only and the original Specialization Score as the focal predictor. The Specialization Score effect is H2a: b = 0.10, SE = 0.10, 95% CI [−0.10, 0.30], p = .320; H2b: b = 0.13, SE = 0.08, 95% CI [−0.03, 0.29], p = .102. Δ−2LL for the baseline models refers to the comparison with the respective null model. * p < .05. ** p < .01. *** p < .001.

**Table S9.3.** Distributional Characteristics of the Hypothesis 2 Outcomes (2,796 Observations).

| **Statistic** | **Perceived ER effectiveness** | **Interaction evaluation** |
| --- | --- | --- |
| M (SD) | 5.58 (1.22) | 5.73 (1.03) |
| Proportion of ratings at the scale maximum (= 7) | 23.0% | 13.1% |
| Proportion ≥ 6.5 | – | 29.4% |
| Cronbach’s α of the four-item composite | – | .90 |

*Note.* The four-item composite applies to interaction evaluation only; perceived ER effectiveness was assessed with a single item. The multilevel generalizability coefficients of the composite are reported in Table S10.2.

**S10. Psychometrics of the Adapted Scales**

**Table S10.1.** Internal Consistencies in the Present Sample (N = 106).

| **Scale** | **Cronbach’s α** | **McDonald’s ω (total)** |
| --- | --- | --- |
| FS-D (8 items, two-week adaptation) | .90 | .90 |
| SELSA Social Loneliness (14 items, German adaptation, two-week timeframe) | .92 | .93 |

*Note.* No item-level missing data occurred (all items were mandatory in the m-Path application). Item-level statistics and the output of an exploratory factor analysis of the adapted SELSA items are available in the OSF repository; given the sample size (N = 106), the factor-analytic results should be interpreted with caution.

**Table S10.2.** Multilevel Generalizability Coefficients for the Four-Item Interaction Evaluation Composite.

| **Coefficient** | **Value** | **What it quantifies** |
| --- | --- | --- |
| R kRn | > .99 | Reliability of between-person differences in the composite when averaged over all of a participant’s occasions (time nested within persons). |
| R cn | .85 | Reliability of within-person, occasion-to-occasion variation in the composite, averaged over items. This is the coefficient relevant for the Level-1 analyses of Hypothesis 2. |
| R kR | .76 | Generalizability of the average across occasions, random time effects. |
| R 1R | < .01 | Generalizability of a single occasion across items, random time effects. |
| R c | .85 | Generalizability of change, fixed occasions and items. |
| R kF | .95 | Reliability of the average of all ratings across items and occasions, fixed time effects. |
| Cronbach’s α | .90 | Internal consistency of the four items across all 2,796 observations. |
| Cronbach’s α (person means) | .94 | Internal consistency of the four items across person means. |

*Note.* Coefficients from multilevel.reliability() in the R package psych (Revelle, 2025), estimated with the lme option; 11,184 item responses from 2,796 occasions nested in 106 participants. The corresponding lme variance components are .24 between persons (17%), .70 between occasions within persons (49%), and .48 residual (34%). The ANOVA-based decomposition returned by the same function is unstable for this unbalanced design (it yields a negative variance estimate for the time effect) and is therefore not reported; the lme-based components are used throughout. The very high value of R kRn reflects the large number of occasions per participant and must not be interpreted as the reliability of a single momentary rating.

**S11. Interaction Modality: Frequencies and Three-Level Sensitivity Model**

**Table S11.1.** Strategy × Modality Cross-Tabulation of the Reported Contacts.

| **Strategy** | **In-person** | **Synchronous virtual** | **Text-based** |
| --- | --- | --- | --- |
| Problem Solving | 272 | 64 | 152 |
| Encouragement | 168 | 25 | 41 |
| Reappraisal | 168 | 25 | 61 |
| Humor | 583 | 73 | 110 |
| Distraction | 705 | 57 | 192 |
| Touch | 75 | 9 | 16 |

*Note.* Synchronous virtual = phone or video call (contacts labeled “other” were classified as synchronous virtual). Notably, 25 touch-motive reports occurred in non-physical contacts (9 synchronous, 16 text-based). Because the motive item assessed the benefit participants hoped to obtain, such reports are interpretable as a desire for closeness expressed in remote contact; they underscore that the item captured interaction motives rather than enacted regulation behavior (see Limitations, main text).

Model results (three-level modality). Relative to in-person contact, interaction evaluations (H2b) were lower for synchronous virtual contact (b = −0.27, p < .001) and markedly lower for text-based contact (b = −0.65, p < .001); the leave-one-out partner–motive residual remained non-significant (H2a: b = −0.03, p = .394; H2b: b = −0.02, p = .319). The model uses the primary random-effects structure.

Exploratory probe (masking by regulatory demand). The NSI was essentially unrelated to the presence of acute stressors during the study period (*r* = −.10) and negatively related to the number of reported interactions (*r* = −.18), consistent with the mechanical dependence addressed by the covariate-adjusted models (see the note to Table S8.2).

**References**

Debrot, A., Schoebi, D., Perrez, M., & Horn, A. B. (2013). Touch as an interpersonal emotion regulation process in couples’ daily lives: The mediating role of psychological intimacy. Personality & Social Psychology Bulletin, 39(10), 1373–1385. <https://doi.org/10.1177/0146167213497592>

Gross, J. J. (2002). Emotion regulation: Affective, cognitive, and social consequences. Psychophysiology, 39(3), 281–291. <https://doi.org/10.1017/s0048577201393198>

Gross, J. J., & John, O. P. (2003). Individual differences in two emotion regulation processes: Implications for affect, relationships, and well-being. Journal of Personality and Social Psychology, 85(2), 348–362. <https://doi.org/10.1037/0022-3514.85.2.348>

Horn, A. B., & Maercker, A. (2016). Intra- and interpersonal emotion regulation and adjustment symptoms in couples: The role of co-brooding and co-reappraisal. BMC Psychology, 4(1), 51. <https://doi.org/10.1186/s40359-016-0159-7>

Kashdan, T. B., Yarbro, J., McKnight, P. E., & Nezlek, J. B. (2014). Laughter with someone else leads to future social rewards: Temporal change using experience sampling methodology. Personality and Individual Differences, 58, 15–19. <https://doi.org/10.1016/j.paid.2013.09.025>

Kugler, L., & Kuhbandner, C. (2015). That’s not funny! – But it should be: Effects of humorous emotion regulation on emotional experience and memory. Frontiers in Psychology, 6, 1296. <https://doi.org/10.3389/fpsyg.2015.01296>

Marroquín, B., Tennen, H., & Stanton, A. L. (2017). Coping, emotion regulation, and well-being: Intrapersonal and interpersonal processes. In The happy mind: Cognitive contributions to well-being (pp. 253–274). Springer, Cham. <https://doi.org/10.1007/978-3-319-58763-9_14>

Niven, K., Totterdell, P., & Holman, D. (2009). A classification of controlled interpersonal affect regulation strategies. Emotion, 9(4), 498–509. <https://doi.org/10.1037/a0015962>

Revelle, W. (2025). psych: Procedures for psychological, psychometric, and personality research [Computer software]. Northwestern University. <https://CRAN.R-project.org/package=psych>

Suvilehto, J. T., Glerean, E., Dunbar, R. I. M., Hari, R., & Nummenmaa, L. (2015). Topography of social touching depends on emotional bonds between humans. Proceedings of the National Academy of Sciences of the United States of America, 112(45), 13811–13816. <https://doi.org/10.1073/pnas.1519231112>

Wolgast, M., & Lundh, L.-G. (2017). Is distraction an adaptive or maladaptive strategy for emotion regulation? A person-oriented approach. Journal of Psychopathology and Behavioral Assessment, 39(1), 117–127. <https://doi.org/10.1007/s10862-016-9570-x>

Wong, Y. J., Li, P. F. J., Cheng, H.-L., & McDermott, R. C. (2025). The anatomy of encouragement: Addressing the what, why, when, who and for whom of encouragement messages. Journal of Prevention and Health Promotion, 6(4), 560–590. <https://doi.org/10.1177/26320770251330870>
